# Host genotype controls ecological change in the leaf fungal microbiome

**DOI:** 10.1371/journal.pbio.3001681

**Published:** 2022-08-11

**Authors:** Acer VanWallendael, Gian Maria Niccolo Benucci, Pedro Beschoren da Costa, Linnea Fraser, Avinash Sreedasyam, Felix Fritschi, Thomas E. Juenger, John T. Lovell, Gregory Bonito, David B. Lowry

**Affiliations:** 1 Department of Plant Biology, Michigan State University, East Lansing, Michigan, United States of America; 2 Ecology, Evolution, and Behavior, Michigan State University, East Lansing, Michigan, United States of America; 3 Great Lakes Bioenergy Research Center, East Lansing, Michigan, United States of America; 4 Department of Plant, Soil, and Microbial Sciences, Michigan State University, East Lansing, Michigan, United States of America; 5 Genome Sequencing Center, HudsonAlpha Institute of Biotechnology, Huntsville, Alabama, United States of America; 6 Division of Plant Sciences, University of Missouri, Columbia, Missouri, United States of America; 7 Department of Integrative Biology, University of Texas, Austin, Texas, United States of America; Duke University, UNITED STATES

## Abstract

Leaf fungal microbiomes can be fundamental drivers of host plant success, as they contain pathogens that devastate crop plants and taxa that enhance nutrient uptake, discourage herbivory, and antagonize pathogens. We measured leaf fungal diversity with amplicon sequencing across an entire growing season in a diversity panel of switchgrass (*Panicum virgatum*). We also sampled a replicated subset of genotypes across 3 additional sites to compare the importance of time, space, ecology, and genetics. We found a strong successional pattern in the microbiome shaped both by host genetics and environmental factors. Further, we used genome-wide association (GWA) mapping and RNA sequencing to show that 3 cysteine-rich receptor-like kinases (crRLKs) were linked to a genetic locus associated with microbiome structure. We confirmed GWAS results in an independent set of genotypes for both the internal transcribed spacer (ITS) and large subunit (LSU) ribosomal DNA markers. Fungal pathogens were central to microbial covariance networks, and genotypes susceptible to pathogens differed in their expression of the 3 crRLKs, suggesting that host immune genes are a principal means of controlling the entire leaf microbiome.

## Abstract 2

Leaf fungal microbiomes can strongly influence host plant success. Monitoring the leaf fungal microbiome of switchgrass over time shows microbial ecological succession, and reveals the host plant genes that influence community-wide changes.

## Introduction

Microbial communities perform essential functions for their host organisms in all branches of life. In some systems, hosts can tightly control the microbes with which they form symbioses [1,2]. In others, the composition of the microbiome is more governed by ecological interactions such as the order of species arrival or abiotic conditions during colonization [3,4]. A key goal of microbial evolutionary ecology is to determine how both host and nonhost factors influence microbiome assembly [5], particularly in natural settings where host influence is more challenging to study.

Communities that colonize available niches in the process of succession follow certain predictable ecological patterns. Early-arriving species are typically those with effective long-range dispersal, while the climax community is dominated by species that can more effectively use resources under competition [6]. While these broad patterns are generalizable, the composition of any particular successional community depends greatly on both the habitat colonized and interspecific interactions such as priority effects, where the order of arrival of taxa governs the success of later arrivals [7,8]. While most successional theory is based on studies in macro-scale organisms, the principles of succession are evident in microbial communities as well, but on a more rapid timescale [9–11].

In the case of microbiomes, host factors governing microbial succession must also be considered. Since the composition of the microbiome can greatly impact host fitness, it can be evolutionarily beneficial for the host to play a role in the successional process, encouraging mutualist colonization while dispelling pathogens as the community assembles. Hosts express genes that influence colonizing microbes through several means, including immunity, morphological adaptations [12], and chemical exudation [13]. While the immune system is often effective at preventing detrimental infections, immune receptors may recognize and exclude beneficial microbes if elicitors are structurally similar to a pathogen, so specific immunity can have wider impacts on the microbiome [14]. Hosts require finely calibrated mechanisms for attracting beneficial microbes without attracting pathogens in a constant coevolutionary push and pull.

The phyllosphere microbiome, consisting of the microbes on and inside the plant leaf, comprises diverse taxa that impact plant health and productivity [15–18]. Leaf fungi in particular are common plant pathogens [19], but nonpathogenic taxa may perform beneficial functions for the host, including nutrient uptake and pathogen antagonism [20–25]. Since the phyllosphere microbiome of perennial plants is reassembled at the start of each growing season in freshly sprouted tissues, [26,27] it may show similar patterns to macro-scale secondary successional communities. Recent research has shown that host control of the leaf microbiome is often governed by numerous loci of small effect directly impacting relatively few microbes [28–30].

We hypothesized that the phyllosphere fungal microbiome develops seasonally as a successional community controlled by environmental factors, host genetics, and interspecific fungal–fungal associations. We used amplicon sequencing to compare the relative importance of these factors in the phyllosphere fungi of a replicated diversity panel of switchgrass (*Panicum virgatum* [31]). We tested whether communities change directionally and whether the trajectory of succession differed across switchgrass genetic subpopulations and across different growing sites. Additionally, we sought to uncover whether specific genetic loci underlie host control of the microbiome through genome-wide association study (GWAS) and RNA sequencing analyses. Finally, we investigated the roles of specific fungal taxa in the microbiome through network analysis. Specifically, we aimed to determine whether known switchgrass leaf pathogens [32] covary with nonpathogenic symbionts, or are peripheral to microbial communities.

## Results

### Succession varies across host subpopulations and planting sites

Switchgrass is a highly genetically diverse perennial grass native to North America, and both plant traits and switchgrass–microbe interactions vary across its range [31–33]. We leveraged this diversity to assess the difference in microbial communities across the 3 main switchgrass subpopulations by randomly selecting 106 genotypes from a diversity panel [31] planted at our focal site, the Kellogg Biological Station (KBS), Michigan, United States of America. Of these, 28 genotypes were from the Midwestern subpopulation, 38 from the Atlantic, 31 from the Gulf, and 9 showed signs of admixture between groups (Intermediate). These subpopulations differ in morphological and ecological characteristics, so we expected that fungal succession would differ as well across subpopulations. Switchgrass subpopulations correspond roughly to 3 morphological ecotypes: Midwest genotypes are mostly Upland, Gulf are Lowland, and Atlantic mostly Coastal [31]. Since the samples used in this study largely followed this pattern, subpopulation differences can also be considered ecotypic differences. We examined succession over time by sampling leaf tissue from each plant at 5 time points, then sequencing the internal transcribed spacer (ITS) region of the phyllosphere-associated fungi in and on the leaf. After quality filtering, we clustered 47.8 million ITS reads to 6,756 fungal operational taxonomic units (OTUs) that varied across genotypes and over time.

**Fig 1:**
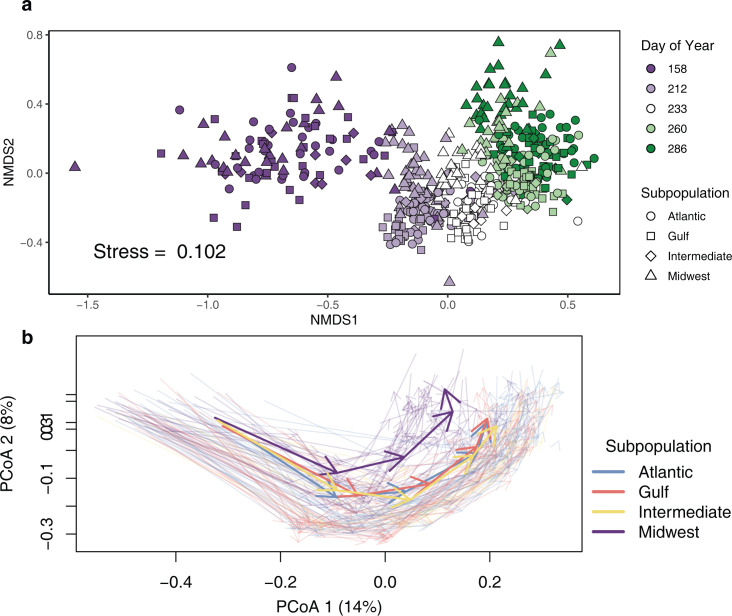
Structural and successional change in the leaf phyllosphere community shown by 2 methods. Each point represents an individual plant sampled from the experimental plot at KBS, Michigan. **(A)** NMDS. Dates are shown as DOY. Points are colored by DOY, and switchgrass subpopulations as shapes. **(B)** Trajectory plots of principal coordinates of community distances. Transparent arrows represent individual switchgrass genotypes sampled over the 5 dates shown in (A), and colors show genetic subpopulations. Solid colored arrows show mean subpopulation trajectories. Data underlying this figure can be found in S1 Data. DOY, day of year; KBS, Kellogg Biological Station; NMDS, nonmetric multidimensional scaling.

To determine the directionality of successional changes in the microbiome, we visualized community differences with nonmetric multidimensional scaling (NMDS). NMDS accurately preserved sample distances in reduced dimensions (Stress = 0.102; S1 Fig) and revealed clear temporal community structure. NMDS1 clustered closely with the date of collection, while NMDS2 clustered more with host genetic subpopulation (Fig 1A). Notably, the first sampling date was highly distinct from the later time points, showing greater variation within that time point, as well as divergence from later time points (Fig 1). To explore the statistical significance of patterns of succession, we used permutational multivariate analysis of variance (PERMANOVA). Both sampling date (day of year, DOY) and subpopulation had significant effects on community structure, but differed greatly in their explanatory power (Table 1). At the focal site, KBS, collection date (DOY) explained the greatest amount of variation (19.4%), followed by genetic subpopulation (5.7%), and there was a significant date-by-subpopulation interaction (2%). To assess the impact of disease on leaf microbiome, we performed a separate test with the subset of samples for which we were able to collect both infected and symptomless leaves. We restricted permutations within individual plants to perform the equivalent of a paired test of infection effects, resulting in a significant infection term that explained 7.79% of the variation in community distance (*p* < 0.001).

**Table 1:** PERMANOVA of a Bray–Curtis community dissimilarity matrix for genotypes at the focal site (*n* = 106) and those replicated across sites (*n* = 8).

KBS sequential					
	Df	SumOfSqs	R^2^	F	Pr (>F)
**DOY**	**1**	**15.6**	**0.194**	**143.64**	**0.001**
**Subpopulation**	**3**	**4.6**	**0.057**	**14.03**	**0.001**
**DOY:Subpopulation**	**3**	**1.6**	**0.02**	**4.97**	**0.001**
Residual	538	58.5	0.728		
Total	545	80.3	1		

Multisite sequential					
	Df	SumOfSqs	R^2^	F	Pr (>F)
**Site**	**3**	**4.6**	**0.296**	**12.14**	**0.001**
**DOY**	**1**	**1.1**	**0.072**	**8.88**	**0.001**
**Subpopulation**	**2**	**0.7**	**0.046**	**2.82**	**0.001**
**DOY:Site**	**3**	**1.4**	**0.090**	**3.70**	**0.001**
Residual	61	7.8	0.496		
Total	70	15.6	1.000		

DOY is sampling date, subpopulation indicates genetic group, and site indicates planting site. Terms are shown with sequential effects. All terms were significant with α < 0.05.

DOY, day of year; KBS, Kellogg Biological Station; PERMANOVA, permutational multivariate analysis of variance.

In order to directly test the differences in succession across subpopulations, we modeled changes in the multidimensional representation of fungal communities as directional trajectories [34]. Across the season, fungal communities on individual plants showed parallel changes over time, with almost no reversals to earlier community states (Fig 1B), strongly indicating a successional pattern. Switchgrass genetic subpopulations differed in both mean trajectory length (df = 3, F = 2.786; *p* = 0.0453) and mean overall direction (df = 3, F = 3.677; *p* = 0.0151). While little subpopulation difference is evident at the beginning of the season, climax fungal communities were markedly different in the Midwestern population, which showed the greatest divergence from others in trajectory direction (Fig 1B, Midwest-Atlantic; Tukey honest significant difference [HSD] = 0.013, *p* = 0.050). This provided initial evidence that, while fungal dispersal is similar across plant subpopulations, host plants influence the climax state of fungal communities. Further, we tested for differences between genotypes that were presenting rust-associated symptoms and genotypes that were not presenting symptoms. Infected and symptomless plants changed along parallel trajectories that differed in length (df = 1, F = 5.274, *p* = 0.0238), but not in directionality (df = 1, F = 0.652, *p* = 0.421).

**Fig 2:**
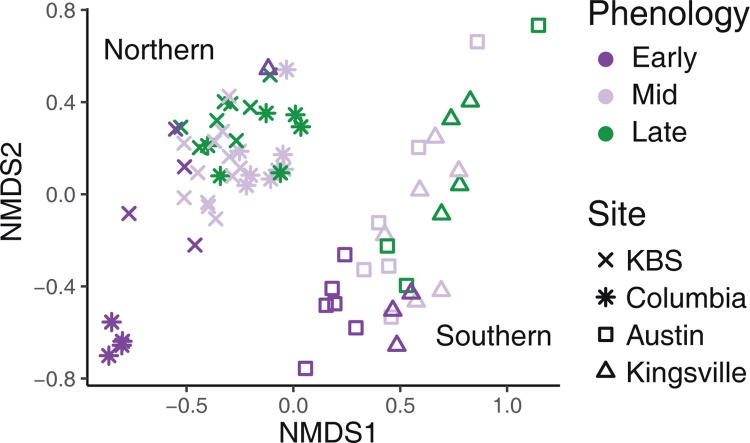
Site-specific changes in microbial communities shown by NMDS. Genotypes were sampled at 4 sites. From north to south: KBS, Michigan; Columbia, Missouri; Austin, Texas; and Kingsville, Texas. Northern sites are shown by symbols, and southern by open shapes. Color indicates phenological stage sampled, “Early” samples were taken just after emergence, “Mid” samples were taken during seed development, and “Late” samples were taken after senescence began. Data underlying this figure can be found in S2 Data. NMDS, nonmetric multidimensional scaling.

Fungal microbiomes can be greatly influenced by environmental factors in addition to host factors. Therefore, we compared succession across environments by selecting a subset of 8 plant genotypes replicated in 3 additional sites across a latitudinal gradient in the USA. From north to south, these field sites were Columbia, Missouri; Austin, Texas; and Kingsville, Texas (S2 Fig). We sampled each site at 3 time points, standardized by phenology to account for seasonal differences across sites (S3 Fig). At most sites, collection date correlated with both NMDS1 and NMDS2 (Fig 2; stress = 0.103). However, the northern and southern sites were divided on a diagonal line orthogonal to collection date. The northern sites KBS and Columbia, Missouri formed one cluster, while the southern sites, Austin, Texas and Kingsville, Texas formed another (Fig 2). Differences across sites accounted for 29.6% of the variation in community dissimilarity across sites, but sampling date and subpopulation also structured the community to a lesser extent (Table 1). While succession may show temporal patterns in southern sites, the composition of fungal communities on leaves is largely distinct. A significant site:sampling date interaction term indicates that succession differs across sites, but additional samples may be needed to fully understand this pattern.

### Host genetic subpopulations support divergent fungal communities

**Fig 3:**
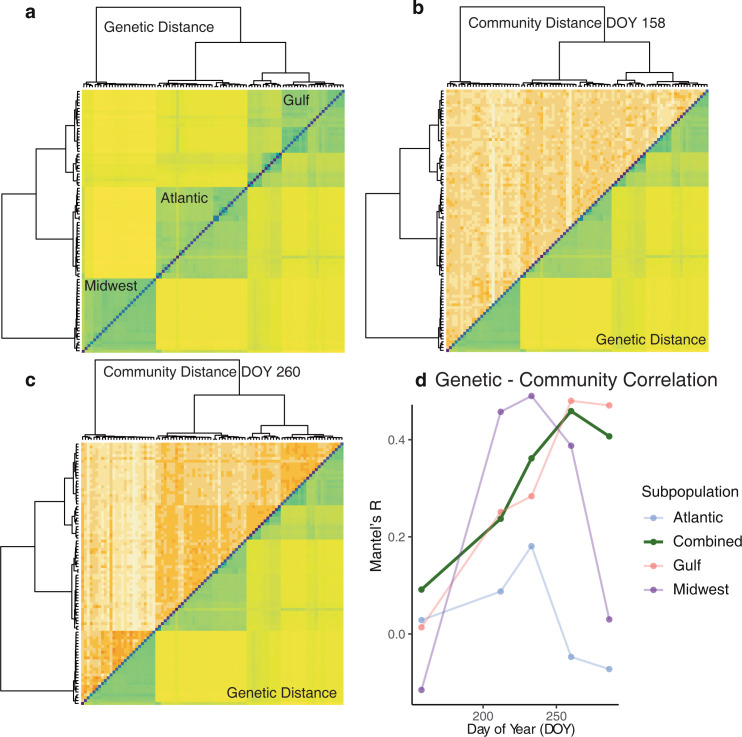
Genetic and fungal community pairwise distance matrices at KBS, Michigan. **(A)** Pairwise genetic distance (π) for all samples. Samples are ordered by hierarchical clustering. **(B, C)** Pairwise community distance (Bray–Curtis) for all samples, shown in the same order as genetic distances, for 2 sampling times, DOY 158 and DOY 260. Other sampling times shown in Supp. (D) Values of Mantel’s r shown indicate correlation between distance matrices for genetics and fungal communities at each time point, with subsetted subpopulations shown as faint lines. *p* < 0.01 for all tests in the combined populations. Data underlying this figure can be found in S3 Data. DOY, day of year; KBS, Kellogg Biological Station.

Beyond differences at the level of subpopulations, we expected that within-subpopulation genetic differences would impact fungal diversity. To further examine genetic differences over time, we compared host genetic distances to fungal community differences between plants at the focal site, KBS. Genetic distances, calculated as Nei’s distance using 10.2 million single nucleotide polymorphisms (SNPs) [35], revealed that host population genetic structure largely matched the 3 major switchgrass genetic groups observed previously: “Gulf,” “Atlantic,” and “Midwest” [31] (Fig 3A). These 3 subpopulations are deeply diverged and serve as discrete gene pools within which we tested for host-driven fungal community divergence. Fungal community distances, calculated as Bray–Curtis community dissimilarity, varied across sampling dates, but largely recapitulated the genetic structure of switchgrass (Fig 3B and 3C). Notably, Mantel tests showed that fungal community structure was most closely correlated with host genetic structure at DOY 260, when most plants had set seed (r = 0.453), but declined as senescence progressed (Fig 3D). While we anticipated some degree of genetic influence, subpopulations were even more highly structured than expected, with almost half of the variation in fungal community distance explained by genetic distance when plants are setting seed (DOY 260). To confirm this pattern, we estimated pseudo-heritability values for community structure using the kinship matrix as a random effect in mixed models. Overall, there was weak heritability for variation on the second NMDS axis (*H*^2^ = 0.132 ± 0.043), but this may be attributable to high variation across time points (DOY 158: 0.120 ± 0.188, DOY 212: 0.164 ± 0.146, DOY 233: 0.184 ± 0.733, DOY 260: 0.950 ± 0.176, DOY 286: 0.913 ± 0.352).

**Fig 4:**
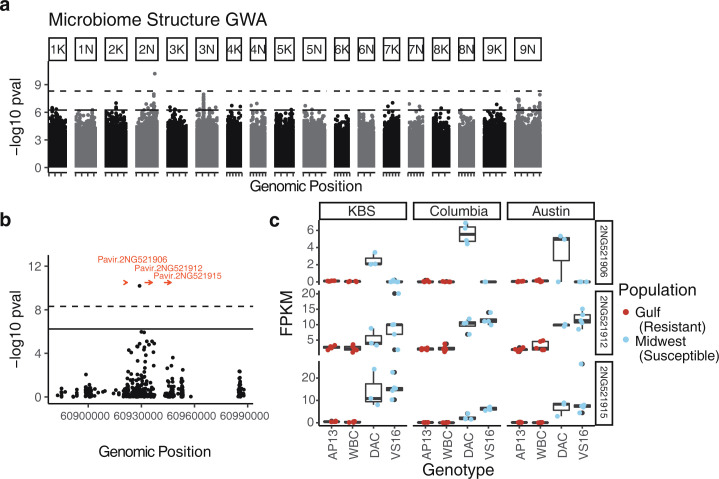
Genetic associations with microbiome structure. **(A)** GWAs of microbiome structure (NMDS2 at DOY 260). The lower solid line shows the 5% FDR threshold, and the upper dashed line shows the Bonferroni-adjusted alpha threshold for SNPs associated with the microbiome. **(B)** Outlier region on Chromosome 2N with nearby genes shown in red. **(C)** Expression-level differences for genes shown in (B). Leaf tissue for these samples was collected as part of a different study, performed at 3 of the same sites we used. FPKM are scaled differently in each gene facet. Data underlying this figure can be found in S4 Data. DOY, day of year; FDR, false discovery rate; FPKM, fragments per kilobase of transcript per million mapped reads; GWA, genome-wide association; NMDS, nonmetric multidimensional scaling; SNP, single nucleotide polymorphism.

Such tight host–microbiome genetic diversity associations imply a genetic basis of influence on fungal community dynamics by their plant hosts. To identify the genetic loci that might underlie this pattern, we calculated genome-wide associations (GWAs) for microbiome structure. We used the second NMDS axis at DOY 260 from the above analysis (Figs 1 and S4) to represent microbiome structure, since it showed the greatest clustering with subpopulation (Figs 1A and 3C, additional time points in S5 Fig) and controlled for large-scale host genetic structure by including a single variate decomposition of pairwise genetic distance as a covariate in the linear models. We found several loci associated with the phenotype at a 5% false discovery rate (FDR), but the GWA showed an excess of low *p*-values (quantile–quantile plot: S6 Fig) so we used a more conservative Bonferroni-corrected threshold to identify significant SNPs (Fig 4A). This threshold revealed only 1 SNP on chromosome 2N significantly associated with microbiome structure, Chr02N_57831909. This SNP is closely linked to several genes in the switchgrass v5.1 genome annotation (Fig 4B and 4C). The 3 closest genes are homologous to receptor-like kinases (RLKs) annotated in the closely related *Panicum hallii* (2 copies of *cysteine-rich receptor-like protein kinase 6; XP_025800480*.*1 and XP_025800481*.*1*, and 1 copy of *cysteine-rich receptor-like protein kinase 10; XP_025801715*.*1)*. This class of RLKs is diverse in plants, but is known to contain many immune receptors [36], indicating a potential role for these genes in host control of fungi.

We corroborated the importance of these candidate genes by comparing their expression levels in divergent genotypes at the 3 of the 4 sites where phyllosphere experiments were conducted, KBS, Columbia, and Austin. In each site, we sequenced leaf tissue RNA from multiple biological replicates (*n* ≥ 3) from 4 genotypes: 2 that are typically more susceptible to leaf fungal pathogens (Midwest upland VS16 and DAC) and 2 that typically are more resistant (Gulf lowland WBC and AP13) [32]. Consistent with host-gene driven variation in fungal community assembly, all 3 candidate genes were much more highly expressed in susceptible than resistant genotypes (Wald tests; Table 2). These differential genotype-specific patterns of expression were very similar across planting sites (likelihood ratio test for ecotype ✕ site interaction, *p* = 0.354).

**Table 2:** Wald test for expression differences in 3 candidate genes between Midwest (more susceptible) and Gulf (more resistant) populations.

Wald test					
	Base mean	Log fold change	SE	W	*p*-value
Pavir.2NG521906	12.91	3.22	0.81	3.95	7.89E-05
Pavir.2NG521912	313.63	1.99	0.16	12.25	0.00E+00
Pavir.2NG521915	189.09	5.33	0.30	17.94	0.00E+00

Data underlying this table can be found in S1 Data.

As further confirmation of the importance of the outlier SNP, we selected several genotypes that were not in the original study (*n* = 20) containing differing alleles of the outlier SNP. We used the same protocols as the first round of sampling, but only sampled at one location at 2 time points, KBS, Michigan. Fungal microbiomes in these samples conformed closely to our predictions, with allelic variation at the Chr02N_57831909 locus influencing microbiome structure (PERMANOVA F = 1.84, *p* = 0.016, R^2^ = 0.064; S7 Fig). Although this was a smaller subset of samples taken 3 years after the original samples, we were also able to confirm a strong temporal influence on microbiome structure between the 2 time points (PERMANOVA F = 16.80, *p* < 0.001, R^2^ = 0.293; S7 Fig). In addition, we sequenced the fungal large subunit (LSU) in these samples to exclude any primer bias against any taxonomic group. LSU sequences showed a similar temporal pattern to fungal ITS and were structured with the Chr02N_57831909 locus in a strikingly similar pattern to ITS (PERMANOVA F = 2.66, *p* = 0.001, R^2^ = 0.086; S7 Fig). For both amplicons, population structure as indicated by PCs 1 and 2 of the genomic SVD explain some variation in the microbiome, but not as much as allelic variation at Chr02N_57831909. Together, these results suggest that the Chr02N_57831909 locus has a stable and deep influence on the microbial community.

### Yeasts, pathogens, and mycoparasites are core phyllosphere microbiome members

Given the large differences in leaf pathogen susceptibility across switchgrass subpopulations, we sought to determine the influence of pathogenic fungi on other members of the fungal microbiome. We examined the taxonomic relationships of the 7392 OTUs in our dataset through a hybrid method that compares matches across fungal databases and BLAST (Basic Local Alignment Search Tool) hits [37]. We identified 6,756 OTUs as fungi, and excluded 633 plant, and 3 metazoan OTUs. We performed NMDS and PERMANOVA analyses using the full fungal community, but focused our taxon-specific analyses on OTUs at the focal site that were present at high occupancy across time and showed relatively high abundance, often referred to as the “core” microbiome [38]. This group consisted of 128 OTUs, the majority of which were Dothideomycetes (43.5%) and Tremellomycetes (28.7%, S1 Table). We assigned each of the core OTUs to a functional guild when possible using published literature (S1 Table). Of the core group, 23 OTUs were grass pathogens, and 9 were documented pathogens of other plants. Four were known mycoparasites, fungi that prey upon other fungi. Three were generalist decomposers or had an unclear functional guild, and the remaining 52 were yeasts or yeast-like fungi. Compared to fungal species in soil, these taxa were especially enriched for grass pathogens and yeasts and contained much fewer saprophytes [39].

**Fig 5:**
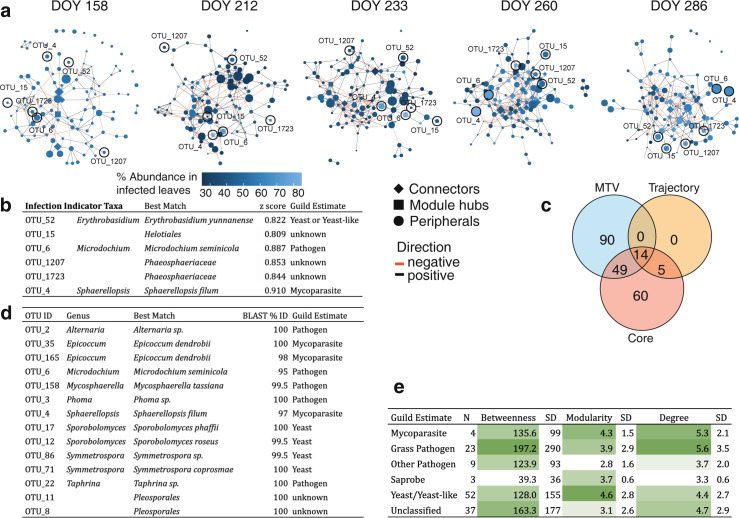
Network analysis of core OTUs. **(A)** Covariance networks of core OTUs over time. Nodes are colored by each OTU’s relative abundance in infected leaves with visible symptoms. The shape of the node denotes network position, defined by Zi-Pi ratio. Edges are colored by the covariance sign. **(B)** Infection indicator taxa, including best taxonomic match and z-score for indicator analysis. **(C)** Number of OTUs identified as important by several methods: MTV-LMM analyses that indicate time-dependent OTUs, OTUs that impact the successional trajectory, and core OTUs with high occupancy-abundance. **(D)** Taxonomic information for the 14 OTUs identified in all 3 analyses in (C). Best match denotes the lowest taxonomic level confidently identified for each OTU using BLAST. Guilds were estimated based on published studies, references are in S1 Table. **(E)** Network statistics for fungal guilds, calculated as mean values across all time points, with (SD. Data underlying this figure can be found in S5 Data. DOY, day of year; MTV-LMM, microbial temporal variability mixed linear model; OTU, operational taxonomic unit; SD, standard deviation.

To investigate how these functional guilds associate, we built covariance networks using OTU relative abundances at each time point (Fig 5A). We summarized network statistics across functional guilds to show that known grass pathogens are central to covariance networks, with high betweenness centrality (extent to which a node lies on paths connecting other nodes) and degree (overall number of connections; Fig 5E). Standard deviation was high within this group, however, reflecting seasonal and within-group differences. Yeasts, in contrast, showed higher modularity (compartmentalization; Fig 5E). This indicates that, while yeasts are overall more speciose in the core microbiome, they covary less with the rest of the microbial community than pathogens. Since yeasts are thought to be mostly commensal inhabitants of the outer leaf surface [40], this difference may reflect their ecological or spatial niche.

In addition to varying among functional groups, OTU covariance also significantly changed over time as supported by the bootstrap-permutation based network comparisons between each sampling point (Fig 5A and S6 and S2 Tables). To identify positive or negative covariance temporal patterns within network members, we generated a Class-level heatmap showing the proportion of edges linking OTUs within or between each Class at each time point (S8 Fig). Due to the high proportions of Dothideomycetes (mixed guilds) and Tremellomycetes (yeast) in the core, the majority of edges at every time point were within (38.3% to 48.6%) and between (9.9% to 14.5%) OTUs in these classes. While the proportion of positive edges maintained more or less stable with time between OTUs in the Dothideomycetes (from 20.0% to 23.9%) and Tremellomycetes (from 23.0% to 17.4%) or within the 2 classes (from 3.6% to 2.8%), negative edges between classes increased from DOY 158 (6.7%) to DOY 233 (9.7%) and DOY 286 (11.7%). This may indicate competition for host resources between these 2 classes of fungi, resulting in more spatially heterogeneous distributions in the late season.

While patterns in this core group reflected major changes in the fungal microbiome, we used several alternate methods to identify important OTUs. In addition to the core, we modified trajectory analyses (Fig 1B) by computationally removing each OTU from the analysis and calculating the change in the overall community trajectory [34]. Nineteen OTUs significantly impacted trajectories when they were removed, all of which overlapped with the core group (Fig 5C). To examine priority effects, we used microbial temporal variability mixed linear models (MTV-LMMs), which identify taxa for which variation in earlier time points explains variation in later points [41]. Of the 153 OTUs we found in this analysis, 49 overlapped with the core, and 14 with both the core and trajectory analysis (Fig 5C). The 14 OTUs that were identified as important using all 3 methods (Fig 5C and 5D) included taxa from several putative functional guilds, including yeasts, pathogens, and mycoparasites. Network connections confirmed mycoparasitic interactions; we found a negative relationship between putative plant pathogens *Mycosphaerella tassiana* and *Microdochium seminicola* versus mycoparasitic *Epicoccum dendrobii*. In addition, we used indicator species analysis to identify OTUs that were overrepresented in leaves with fungal disease symptoms (Fig 5A). Although the major fungal pathogen of switchgrass, *Puccinia novopanici*, was not identified as a core taxon, indicator species analysis showed that putative mycoparasite *Sphaerellopsis filum* is present in the core and significantly associated with fungal infection symptoms (OTU_4; Fig 5A).

Our analyses mostly identified the taxa that were abundant across samples. Rare taxa can be important in microbial community functioning [42], but their role in overall ecological patterns is less clear and more challenging to study. Therefore, we only examined rare taxa that we expected a priori to play an important ecological role. *Claviceps* species were present in 119/760 samples, and were more highly abundant in the early season. *Claviceps* species produce alkaloid compounds that deter grazing [43], so this endophyte may play a role in protecting young grass shoots. *Metarhizium*, a related genus, was present at low abundances in 43/760 samples in the Columbia, Missouri and KBS, Michigan sites. *Metarhizium* species are insect-pathogenic fungi [44], so may provide a similar protective role.

## Discussion

Our results show strong support for the importance of time, geographic location and host genetics in influencing the switchgrass phyllosphere microbial succession over the growing season. We found evidence for clear successional dynamics that were consistent in direction across growing sites, but were distinct in community composition. Fungal communities were different across host genetic subpopulations, a pattern that may be driven by variation at 3 linked immune receptors. Leaf fungal communities are taxonomically diverse, but a few highly abundant pathogens and yeast species play a disproportionate role in shaping community progression.

Viewing the switchgrass leaf microbial community through the lens of succession allowed us to delineate ecological patterns in these communities. Multidimensional scaling representations of the leaf communities at the focal site revealed a clear clustering by date of collection on the first NMDS axis (Fig 1). This indicates that, as we predicted, date of collection is an important source of variation in the switchgrass leaf fungal community. Further, measuring the trajectories of these communities showed that succession is both directional and deterministic, since no samples showed negative trajectories (reversals of succession) by the end of the season, and most samples followed a similar trajectory (Fig 1B). We observed similar patterns to other studies that show early-season leaves as highly distinct from later time points, perhaps owing to greater influence from soil microbes [16,45]. While the overall shape of trajectories was similar among samples, the Midwestern population deviated from others, particularly in the late season. The Midwestern population is notable since we have previously shown that it is more susceptible to several fungal pathogens such as leaf rust (*Puccinia novopanici*) and leaf spot (*Bipolaris* spp.; [32]; also see [46]) and has on average an earlier phenology than the other population groups [33]. Leaf microbiome relationships are consistently distinct in this population and may be linked to other traits such as cold tolerance that also differ [31,33].

In addition to temporal differences across subpopulations, the composition of fungal leaf communities differed markedly across geographic locations. This may be partially due to seasonality differences across the region we examined. The Kingsville, Texas site did not experience freezing temperatures between 1989 and 2020 (NOAA weather service), so perennial grasses in the region may have living aboveground tissue year-round. Growing season length has been shown as an important factor in governing the abundance and diversity of endophytic fungi [47], so it is unsurprising that we saw large differences across this latitudinal gradient. However, many other factors that influence fungal communities also differ across these sites, including precipitation regime, soil type, and surrounding vegetation, so further work is needed to determine if the growing season is truly the causal factor.

We predicted that fungal communities would be impacted by host genetics as well as location. We found several lines of evidence for genetic control of the leaf microbiome. In addition to examining differing successional trajectories across subpopulations, we tested the covariance of genetic distance and fungal community differences using Mantel correlations. Genetic-fungal community correlations increased until DOY 260, then declined as host senescence began. Mantel tests are inappropriate for some ecological tests and often underestimate *p*-values, but can be useful for exploratory analysis of distance matrices [48]. While there was high variation in our pseudo-heritability estimates, the fact that they mirror temporal patterns in the Mantel tests strengthens the general trend of greater genetic associations in the late season. Previous studies have found similarly high variation in microbiome heritability estimates across time [29], so it is not surprising to see this in our case. Deng and colleagues calculated *H*^2^ for individual OTUs, which ranged from 0 to 0.66 and a Mantel’s correlation of *r* = 0.13 between genetic and microbiome composition in sorghum rhizosphere. This value of *r* is lower than we saw in our study, possibly since it was based on a relatively small subset of samples. When selecting samples for this study, we randomly chose equal numbers of samples from the 2 major switchgrass morphological ecotypes, upland and lowland switchgrass [49] (S2 Fig). Lowland switchgrass, which is more highly represented in Gulf and Atlantic subpopulations, is more resistant to several leaf fungal pathogens [32], so subpopulation differences may be at least partially driven by differences in immunity across these genotypes. Since pathogens such as *Microdochium* and *Alternaria* were among the most abundant taxa in our samples, their differences across subpopulations may have driven overall community differences. In addition to immunity, however, subpopulations differ in other traits that may contribute to fungal colonization differences, such as leaf wax content [50], exudate concentration [51], and phenology [33,49], so microbiome differences may be responding to multiple host plant traits.

### A replicated receptor-like kinase is associated with fungal differences

We found one outlier SNP associated with microbiome structure. While there were several peaks in the Manhattan plot (Fig 5A), our analysis showed a strongly skewed distribution of observed versus expected *p*-value (S6 Fig), indicating a risk of Type I errors. This is probably attributable to the low sample size in this GWAS. The influence of the identified locus is fairly strong, contributing to a clear decrease on NMDS axis 2 when the minor allele is present (MAF = 0.083; S9 Fig). This SNP is not in Hardy–Weinberg equilibrium in switchgrass; we found only one minor-allele homozygote among our samples. This abnormal pattern may be attributable to structural variation at this locus. Switchgrass subpopulations vary widely in genome structure, which may result in alignment mismatches that resemble SNPs, particularly in regions with multiple gene copies [52]. Indeed, this region shows an elevated number of insertions and deletions compared to nearby sections of the 2N chromosome (S10 Fig, data from [31]) and is adjacent to a region dense with repetitive long terminal repeat retroelements (positions 60960000–60980000). Given the confirmatory results for this locus as well as the RNA sequencing results, however, we expect that there is a true phenotypic association with the locus, but that it may be with a structural variant rather than a true SNP.

The 3 nearby genes we identified were replicated variants of a cysteine-rich RLK whose function has not been experimentally verified in *Panicum*. RLKs are one of the largest plant gene families, including over 600 members in *Arabidopsis* [36]. The best studied of these is FLS2, which detects the bacterial flagellin protein and initiates an immune response cascade [53]. The 3 RLKs we identified show high sequence similarity to immune-related cysteine-rich RLKs in *Arabidopsis* and *Oryza*, and contain the “stress-antifungal domain” PF01657, which has been linked to salt stress as well as fungal responses when present in several proteins [54,55]. *Arabidopsis CRK5*, for example, alters defense responses either through resistance to infection or programmed cell death, depending on how the gene is expressed [56], and CRK6 and CRK 14 are involved in the general non–self-response [57]. Related Arabidopsis genes may be the targets of immune repression by bacterial strains [58]. Similarly, the *Oryza* gene *LIL1* (Os07g0488400) improves fungal rice blast resistance when overexpressed [59]. The pattern of these receptors being more highly expressed in pathogen-susceptible plants may seem counterintuitive given that many RLKs are immune receptors. However, this can often occur when pathogens produce effector proteins that target immune receptors [60]. Necrotrophic fungi in particular can benefit by over-inducing plant immune receptors to initiate programmed cell death, [61,62] then feeding on dead plant tissue.

Since allelic variants at this locus have now been associated with variation in the fungal microbiome across several years in natural populations, it may represent a useful target for future research into genetic control of the leaf microbiome. Previous research has shown that microbiome control is often polygenic, with many contributing loci of small effect [28–30]. Uncovering only a single causal locus in this study may be a product of the relatively low sample size; there are more loci associated with microbiome community structure that did not meet the GWAS cutoff, but may contribute to a polygenic architecture for this trait.

### Pathogens and hyperparasites are important in succession

We used several methods to identify important taxa in the phyllosphere community. In several other recent studies, genetic effects on microbiomes appear to be targeted toward particular microbes, with the effects permeating through the community through ecological effects [28,63,64]. We used “core” microbiome analysis to identify OTUs that show high occupancy (presence across multiple samples within a time point [16]). We found that core taxa overlapped well with important taxa identified by MTV-LMMs and trajectory analysis. We can therefore be confident that this group of taxa is influential in the switchgrass phyllosphere (Fig 5). Within this group, we identified several as pathogens, including *Alternaria*, *Mycosphaerella*, *Microdochium*, and *Taphrina*. It is challenging to assign functional guilds to symbiotic fungi, since their benefit or detriment to the host may depend strongly on phenology, abiotic conditions, and ecological interactions [65]. For example, many endophytic fungi are commensal for most of the season, then shift to breaking down plant tissue as the host begins senescence [66]. Others may be weakly pathogenic, but may improve overall host fitness by enhancing nutrient uptake or preventing infection by more effective pathogens [23,67].

Yeasts and yeast-like fungi were also well represented in phyllosphere samples. Yeasts were historically thought to be dominant in the phyllosphere [68], but this may have been an artifact of methods used. Yeasts are more easily culturable than filamentous fungi, and are therefore overrepresented in studies using cultures to measure fungal diversity. The exact relationship between yeasts and plant hosts is not totally clear, but they are typically thought to be mostly commensal symbionts, feeding on small amounts of sugars on the leaf surface [69].

At the focal site, Tremellomycete yeasts and Dothideomycetes dominated the core microbiome and covaried negatively through time. This may be explained by different spatial distributions across samples; Tremellomyctes dominate some samples and Dothideomycetes other samples, but they rarely coexist. Priority effects, wherein early-arriving taxa gain advantage over late-arriving taxa, may therefore play a role in governing colonization in these taxa. Certain Tremellomycete yeasts have been shown to be potential biocontrol agents against pathogens, e.g., *Papiliotrema* spp. [70], and others have been shown to be “hub” taxa or negatively connected with leaf pathogens, e.g., *Dioszegia* spp. [63], both genera with high abundance in our focal site dataset.

One unexpected finding of our taxon-specific analysis was that 2 mycoparasites were identified as important taxa, *Epicoccum* and *Sphaerellopsis*. *Epicoccum* is an ascomycete genus comprising several species with noted antifungal properties [71,72]. The species we identified in this study, *Epicoccum dendrobii*, is being investigated as a biocontrol agent of the pathogenic anthracnose fungus *Colletotrichum gloeosporoides* [73]. Similarly, *Sphaerellopsis filum* has been observed infecting multiple species of *Puccinia* rusts [74,75] and has been shown specifically to reduce switchgrass rust infection [76]. Another surprising finding was that switchgrass rust was not a core species, despite the fact that its disease symptoms are nearly omnipresent each year in the sites we studied [32]. Fungi in the Pucciniaceae family have an ITS sequence that differs substantially from general fungal primers used in this study, which we suspect resulted in reduced amplification of *Puccinia* rusts. We confirmed this suspicion by additionally sequencing the LSU for our confirmatory analysis; using ITS failed to identify any Puccinia rusts in these samples, but LSU identified 10 OTUs as Puccinia present in 18 of 20 samples. There were more than double the Puccinia OTU counts in individuals with the major allele at the focal outlier locus, but a large outlier obscures a reliable statistical pattern. The ubiquity of the *Sphaerellopsis* hyperparasite is a further indication that *Puccinia* may be more prevalent than our sequencing data show, a speculation that is supported by the fact that *Sphaerellopsis* was identified by indicator species analysis as clearly overrepresented in leaves with rust infection. The other OTU most closely associated with disease symptoms is OTU_4, *Microdochium*. While we could find little evidence of known associations between *Puccinia* and *Microdochium* pathogens in published studies, this result suggests that they may have a synergistic effect on host disease.

Bacterial microbiomes may be just as important to leaf function as fungi [16], although ecological patterns may differ in some important ways. We used fungi in this study because they contain more known switchgrass pathogens and may be documented more clearly within leaf tissue without conflict by chloroplast DNA. However, interplay between microbial groups is an essential component to microbiome ecology. Interactions between fungi, bacteria, viruses, microfauna can all mediate impact on hosts. Bacteria [77] and viruses [78] have documented impacts on the functioning of host-dependent fungi in complex and fascinating multilevel interactions. Beyond individual interactions, functional microbiomes in soils require both diverse fungi and bacterial communities, so influence between these groups is impossible to connect to just one single microbe [79].

## Conclusions

Switchgrass leaf fungal communities are highly diverse, and are influenced by both host and environmental factors. Succession occurs each season as communities are assembled through stochastic, environmental, and host-determined processes. Pathogenic fungi play a critical role in the switchgrass leaf phyllosphere community, determining both the trajectory of microbial community development and acting as central nodes in community networks. Host immune genes such as receptor-like kinases control pathogens directly, and the prevalent mycoparasites that prey on them indirectly. The plant genes that control pathogens may therefore provide a principal means by which plants influence changes in their fungal microbiome.

## Materials and methods

### Plant material

We collected switchgrass leaves from a diversity panel established for a separate study [31]. In brief, researchers planted arrays of 732 genotypes of switchgrass clonally replicated at over 15 sites in the USA and Mexico. These genotypes were collected from across the USA, grown in controlled conditions, then clonally split before replanting at all sites. Since 2018, they have been growing in 1.3 m spaced grids with minimal interference for weed control [31]. Researchers used Illumina HiSeq X10 and Illumina NovoSeq6000 paired-end sequencing (2 × 150bp) at HudsonAlpha Institute for Biotechnology (Huntsville, Alabama, USA) and the Joint Genome Institute (Walnut Creek, California, USA) to sequence the genome of each individual. Sequence information for these samples is available on the NCBI SRA: Bioproject PRJNA622568. Lovell and colleagues [31] called 33.8 million SNPs with minor allele frequency (MAF) greater than 0.5%, we used a subset of 10.2 million, which had less than 10% missing data and a MAF greater than 5%.

We used 2 sampling strategies to assess temporal and geographic variation (S3 Fig and S3 Table). For temporal variation, we sampled leaf tissue from 106 genotypes from a diversity panel of switchgrass grown at the KBS, Michigan field site at 5 time points during the 2019 growing season. To assess geographic variation, we collected 8 randomly chosen genotypes representative of switchgrass genetic populations that were replicated in 4 sites that span the geographic range of temperate switchgrass populations KBS, Michigan (42.419, −85.371); Columbia, Missouri (38.896, −92.217); Austin, Texas (30.383, −97.729); and Kingsville, Texas (27.549, −97.881). At each site, we sampled the same 8 genotypes at 3 time points (*n* = 96; S3 Fig). Given that climate varies greatly over this latitudinal range, we standardized collection by phenology rather than date, focusing on switchgrass emergence, flowering, and senescence. Switchgrass genetic variation segregates into 3 main subpopulations that differ greatly in morphology and phenology [31], so we compared fungal community responses over these populations. At all sites, we collected roughly equal numbers of genetic subpopulations (S3 Fig and S3 Table).

For each plant at each time point, we collected 3 leaves. We haphazardly sampled leaves from the middle of the canopy; that is, leaves that were neither close to the base nor the flag leaf. To minimize external contamination, we sterilized gloves between plants, and collected directly into sterile 50mL tubes (UHP tubes, Fisher Scientific, Waltham, Massachusetts, USA). Since we expected that the fungal community would be impacted by the dominant fungal pathogen, leaf rust, we collected 3 leaves with visible rust symptoms as well as 3 symptomless from the same plant when possible (*n* = 35), all of which were used in downstream analyses. We stored tubes on dry ice in the field and while being shipped, then at −80 °C until extraction. For each day of sampling, we also collected a negative control, one tube opened to the ambient air for at least 10s. Samples were shipped overnight on dry ice to Michigan State University (MSU) for processing.

### Amplicon sequencing

We targeted the endophytic (inside the leaf) and epiphytic (on the leaf surface) fungi. To prepare leaves for DNA extraction, we used 4mm biopsy punches (Integra, Princeton, New Jersey, USA) to produce approximately 21 leaf discs pooled across the 3 collected leaves. We sterilized the biopsy punch tool between samples by soaking it overnight in DNAaway (Thermo Fisher Scientific, Waltham, Massachusetts, USA), then washing in DI water. We punched across the leaf blade to fully represent the spatial diversity in leaves. We homogenized leaf tissue by grinding with 2 sterile 3.175 mm stainless steel ball bearings. We placed sealed sterile 1.5 ml tubes with bearings and leaf discs into liquid nitrogen for 10s, then homogenized in a Mini-G bead beater (SPEX sample prep, Metuchen, New Jersey, USA) for 60 s at 1,500 rpm.

To extract DNA, we used QIAGEN Plant Maxi kits, following the manufacturer’s instructions (QIAGEN, Hilden, Germany). This method yields large amounts of plant DNA in addition to fungal, so we used primers for the fungal ITS rDNA region. We performed library preparation for ITS using the ITS1f (5′-CTTGGTCATTTAGAGGAAGTAA-3′) and ITS4 (5′-TCCTCCGCTTATTGATATGC-3′) primers. We used a 3-step amplification process to amplify the target region, add adaptors, and add barcodes for multiplexing as previously reported by Benucci and colleagues [80,81] PCR amplification steps and reagents are included in the supplement (S4 Table). We normalized DNA concentrations using SequalPrep normalization kits (Thermo Fisher Scientific), concentrated libraries using Amicon Ultra 0.5 mL 50K centrifugal filters (EMD Millipore, Burlington, Massachusetts, USA), and removed primer-dimers with Ampure magnetic beads (Beckman Coulter, Brea, California, USA). We randomized samples across plates, then pooled them into 3 libraries for sequencing. We used 4 levels of negative controls to check for contamination at different steps: field controls that were exposed to air at each sampling point, DNA extraction controls, library preparation controls, and a synthetic mock community [82], resulting in a total of 672 samples that included 59 controls. The synthetic mock community contained 12 ITS taxa described in Palmer and colleagues ([82]). We recovered all species present in this community through sequencing.

We sequenced DNA using Illumina MiSeq 300bp paired-end v3 600 cycles kit in the MSU genomics core facility. Sequencing yielded 84.7 M total reads and high-quality data across samples. Across 3 multiplexed libraries, 74.9% of reads had quality scores above 30 (Phred), with an average of 110 K reads per sample (ranging from 110 reads in negative controls to 199 K reads in samples). After quality filtering, 47.8 M reads remained. We used a 97% clustering threshold for identifying OTUs (Operational Taxonomic Units), resulting in 7,963 OTUs across 672 samples.

### RNA sequencing

Vegetatively propagated plants from 4 genotypes were grown in 3 sites (KBS, Michigan; Austin, Texas; and Columbia, Missouri). Two genotypes, AP13 and WBC, fit in the Gulf population group, and are generally resistant to leaf fungal pathogens [32,46]. The other 2 are more closely related to the Midwest population and are more susceptible to leaf pathogens [32,46]. Leaf tissue was harvested and immediately flash frozen in liquid nitrogen and stored at −80°C until further processing was done. Each harvest involved at least 3 independent biological replicates (individual plants). Plants received no supplemental manipulations, so transcript counts represent constitutive expression. High-quality RNA was extracted using standard Trizol-reagent based extraction [83]. RNA-Seq libraries were prepared using Illumina’s TruSeq Stranded mRNA HT sample prep kit utilizing poly-A selection of mRNA. Sequencing was performed on the Illumina HiSeq 2500 sequencer using HiSeq TruSeq SBS sequencing kit. Paired-end RNA-Seq 150-bp reads were quality trimmed (Q ≥ 25) and reads shorter than 50 bp after trimming were discarded. High-quality sequences (404.4 M reads) were aligned to *P*. *virgatum* v5.1 reference genome using GSNAP v.2019-06-10 [84] and counts of reads uniquely mapped to annotated genes (371.8 M reads) were obtained using HTSeq v.0.11.2 [85]. Raw transcript counts are included in S5 Table.

### Bioinformatics

We analyzed amplicon sequences on the MSU HPCC (High-Performance Computing Center) with *qiime* v1.9.1 [86], *fastqc* v0.11.7 [87], *cutadapt* v2.9 [88], *CONSTAX2* [89], and *usearch* v11.0.667 [90]. We demultiplexed sequencing reads using *split_libraries_fastq*.*py* in *qiime1*, then checked for sequencing errors with *fastqc*. We removed barcodes with cutadapt and filtered fastqs with *USEARCH using the* -fastq_filter option with arguments: 1 expected error (-fastq_maxee 1.0), truncation length of 200 (-fastq_trunclen 200), and no unidentified bases (-fastq_maxns 0) [91]. We clustered 97% OTUs with the UPARSE algorithm [92] through the -cluster_otus option, with singletons discarded (-minsize 2). We assigned taxonomy to OTUs using CONSTAX2 [37], which improves OTU identifications using a consensus algorithm between RDP [93], SINTAX [94], and BLAST classifications [37].

### Statistical analyses

We performed downstream analyses in R v4.0.3 [95] using the packages *decontam* [96], *vegan* [97], *phyloseq* [98], *vegclust* [99], and *metagenomeseq* [100]. We used *decontam* to remove contaminants by pruning OTUs that were overrepresented in negative controls, then normalized read depth with functions in the *metagenomeseq* package. Of 7,963 OTUs we clustered, 162 were identified as contaminants and removed from analyses (identifiable contaminants removed are shown in S6 Table). All contaminants showed low abundance and were evenly spread across negative controls, indicating that fungal contamination was minimal in this study.

### Successional dynamics

We visualized community structure using NMDS, which represents the multivariate structure of a community in reduced dimensions (Shepard plot in S1 Fig). NMDS is classically used for dimensionality-reduction in ecological research since it has few assumptions about the underlying data structure, and contains all variance within a limited set of axes, rather than distributed across eigenvectors as in PCoA [101]. We first used a Hellinger transformation to reduce the impact of extreme data points across samples using the *decostand* function, then performed NMDS with *metaMDS*, both in the *vegan* package. We also used permutational analysis of variance to assay the relative importance of various factors in structuring the fungal community implemented through the *adonis2* function in *vegan*. To test the PERMANOVA assumption of multivariate homogeneity of group dispersion (variance from centroids), we used the *betadisper* function in the *vegan* package. Samples at the first sampling time point (DOY 158) had greater dispersion than other time points, so we repeated our analyses with this time point removed. Since the PERMANOVA still showed a strong effect of DOY (*p* < 0.001), we concluded that the significance of this result is not due to heterogeneity of dispersion. To test the community impact of disease symptoms, we performed a separate PERMANOVA test on individuals for which we were able to collect both infected and uninfected leaves. We modeled leaf infection as a block with each individual plant by including infection status as strata in the adonis function in vegan.

To test the importance of historical contingency in temporal community changes, we used a MTV-LMM [41]. The MTV-LMM assumes that temporal changes are a time-homogenous high-order Markov process and fits a sequential linear mixed model to predict the abundance of taxa at particular time points [41]. For each taxon, we calculated “time explainability,” a metric of the degree to which variation in later time points is explained by variation in earlier points [41]. We fit linear mixed models for each OTU present across multiple time points and used a Bonferroni-corrected α to identify taxa that exhibit significant temporal contingency.

In addition, we examined individual and subpopulation-level succession using trajectory analysis [34]. Trajectory analysis transforms multivariate community changes to two-dimensional trajectories, for which parameters of individual community changes can be compared. We calculated mean trajectories for communities in each subpopulation, then used ANOVA to test for trajectory differences across subpopulations. Additionally, we split OTUs between genotypes that showed rust infection symptoms, and those that were symptomless through the whole season, and tested for trajectory differences. We then used a permutational method to discover OTUs that substantially impact succession. We computationally removed each OTU from our dataset, recalculated mean population trajectories, then compared to the original trajectories. We then used the *trajectoryDistances* function in the *vegclust* package to calculate the degree to which removing each OTU altered the overall community trajectory [99].

Conceptualization of ecological communities as trajectories has a long history in ecology [102], but explicit modeling of trajectory parameters has been challenging until relatively recently [34,103,104]. This approach utilizes statistical methods that are typically applied to movement in geometric space [105] to compare movement by a community in multidimensional space [34]. While trajectory analysis has not been applied to changes in microbial communities to our knowledge, other researchers have used the method to understand succession in Amazon forest communities after land-use change [106], and Iberian forests after fires [107]. We visualized these results using PCoA, which allowed us to estimate variance assigned to each axis and to show that our results are robust to mode of dimensionality reduction.

### Genetic associations

To specifically measure the overall microbiome variation explained by genetic structure, we examined the covariation of genetic distance and fungal community distance using Mantel tests. We calculated genetic distance as the number of pairwise SNP differences between each sample (Nei’s distance, π). We used the switchgrass GWAS SNP dataset [35], which features 10.2 million high-confidence SNPs with MAF > 0.05, and calculated distance with the *dist*.*genpop* function in *adegenet* [108]. For microbiome community differences, we used Hellinger-transformed Bray–Curtis distances calculated with the *decostand* function in *vegan*. We performed Mantel tests with 999 permutations using the *mantel* function in *vegan* for each sampling time point at the focal site (KBS). We also confirmed that there was no impact of spatial position within the field by fitting a mixed model for community structure with sampling date as a fixed effect, and field position and genetic kinship as random effects. Using the mmer function in the R package sommer, likelihood ratio tests indicated that models including kinship had improved fit (*p* < 0.001), but fit was not impacted by including a spatial term (*p* = 0.899 [109]). By including a kinship term, we were able to estimate microbiome pseudo-heritability across all dates (same formula as above), and for each sampling date individually using the vpredict function in sommer [109].

To identify specific genetic loci associated with microbiome community structure, we examined GWAs between SNPs and community structure, represented as the second axis from our NMDS analysis (described above). We did not use the first axis, since that clearly clustered with sampling date (Fig 1). We performed GWA using the *switchgrassGWAS* [31] package and the same SNPs as we used in Mantel tests. To correct for population structure, we included the first 10 principal components of a singular value decomposition (SVD) of pairwise genetic distance as a covariate in the linear models. The first 3 SVD axes explain 35.5%, 29.2%, and 9.03% of the variance of the decomposition. The *switchgrassGWAS* package implements linear regression tests for each SNP using the *big_univLinReg* function in *bigstatsR*, which rapidly applies statistical tests across filebacked big matrices using memory mapping [110]. We calculated both a 5% FDR threshold, as well as a Bonferroni-corrected *p*-value threshold to distinguish outlier SNPs.

To verify outlier SNPs, we examined expression-level differences of adjacent genes across divergent genotypes with RNA sequencing data from a separate study (prepublication access through the Department of Energy Joint Genome Institute). We tested for normalized expression differences across switchgrass genotypes using likelihood ratio tests in *DESeq2* [111]. We tested for expression differences across genotypes separately and additionally examined the influence of site using a combined test for genotype ✕ site interaction.

To confirm the variation at the outlier GWAS locus, we repeated leaf collection and amplicon sequencing in a new set of genotypes with differing alleles at the Chr02N_57831909 locus in 2021. We collected 20 plants at 2 time points (June 22 and July 27) at KBS, then extracted DNA and sequenced ITS libraries according to the steps above. In addition, we targeted the fungal LSU using the primer pair LR0R (5′ ACCCGCTGAACTTAAGC 3′) and LR3 (5′ CCGTGTTTCAAGACGGG 3′) to determine if the patterns hold for a different DNA marker region as well, and exclude primer choice biases. We followed the same bioinformatic steps as previously used for ITS, and assessed the impact of alternate alleles on the microbiome using PERMANOVA via the *adonis2* function in *vegan*^*87*^.

### Important taxa

To identify OTUs that are important in structuring the fungal community, we used several complementary methods. In addition to identifying taxa important in temporal dynamics as described above, we also identified “core” taxa [16]. We examined core community taxa using custom scripts [16,112]. Core taxa are defined as those with relatively high occupancy and abundance across all samples and represent those taxa most likely to have a close symbiosis with the host [113]. To calculate the core, we ranked OTUs by frequency, then selected all the OTUs up to the last OTU that adds a 2% increase in beta diversity (Bray–Curtis similarity) between factors (subpopulations and time points) [113]. For the overall core group, we used the intersection between the core across subpopulations and the core across time. Within this core group, we used network analysis implemented in *SpiecEasi* [114] and *igraph* [115] to build covariance networks over time. Nodes in covariance networks can be assigned to 4 possible groups based on the ratio of their within-module (Zi) and between-module connectivity (Pi) [116]. Those with high Zi and Pi are widely connected “network hubs,” those with low Zi and Pi are disconnected “peripherals.” Nodes with high Pi and low Zi are “connectors,” whereas those with high Zi and low Pi are “module hubs” [116]. To statistically assess similarity across networks we adopted a bootstrap-permutation based network comparison method as implemented in the R package *mina*. This approach repeatedly permutes OTUs within a network to evaluate the likelihood of similarity to a second network. In this study, we assessed the degree to which networks at different time points resembled the previous time.

We used indicator species analysis to identify taxa associated with fungal rust disease symptoms. Indicator species analysis identifies particular taxa that are overrepresented based on a factor, and thus represent a useful indicator for that factor [117]. By comparing species present on infected versus uninfected leaves, we could isolate both OTUs associated with disease symptoms and those overrepresented in symptomless leaves.

We further identified an a priori list of taxa that we expected to play important ecological roles in the phyllosphere. These included pathogens that we have previously identified in these plots, including *Puccinia* spp. [32], *Bipolaris* spp., *Tilletia maclaganii* [118], *and Colletotrichum* spp., and taxa with roles in herbivore prevention, including *Claviceps* spp. [43], *Beauvaria* spp., and *Metarhizium* spp. [119].

## Supporting information

S1 Fig Shepard stress plot for NMDS of KBS site. Data underlying this figure can be found in S1 Data. KBS, Kellogg Biological Station; NMDS, nonmetric multidimensional scaling.
(PDF)

S2 Fig Original collection locations for samples. Latitude and longitude are available in S3 Table. The base map uses points from the US Census Bureau, implemented in the maps R package (https://www.census.gov/geographies/mapping-files/time-series/geo/carto-boundary-file.html). Data underlying this figure can be found in S3 Table.
(PDF)

S3 Fig Sampling scheme. Each icon represents one sample taken. We sampled 106 genotypes at 5 time points at the focal site in Hickory Corners, Michigan, and 8 genotypes at the 3 other sites. We sampled roughly equal numbers of each subpopulation throughout. Sites are shown from northern (KBS, Michigan) to southern (Kingsville, Texas). KBS, Kellogg Biological Station.
(PDF)

S4 Fig NMDS plot of the subsetted data used in GWAS analysis. Shapes represent genetic subpopulation. Data underlying this figure can be found in S1 Data. GWAS, genome-wide association study; NMDS, nonmetric multidimensional scaling.
(PDF)

S5 Fig Manhattan plots for additional time points. A. DOY158; B. DOY212; C. DOY233; D. DOY286. Data underlying this figure can be found in S4 Data.
(PDF)

S6 Fig Quantile–quantile plot for microbiome GWAS results showing an excess of observed low *p*-value. Data underlying this figure can be found in S4 Data. GWAS, genome-wide association study.
(PDF)

S7 Fig Fungal community differences across alternate allele states at locus Chr02N_57831909. These results confirm that the locus has a significant impact on the fungal community. The figures at left show NMDS plots of the fungal community at 2 time points sampled in 2021. The right tables show PERMANOVA results for these samples, indicating a significant effect of allele on community structure. Data underlying this figure can be found in S6 Data. NMDS, nonmetric multidimensional scaling; PERMANOVA, permutational multivariate analysis of variance.
(PDF)

S8 Fig Class-level comparison of the proportion of edges linking OTUs within or between each Class in a time point. Column facets show DOY, and row facets show whether connections were negative or positive. Data underlying this figure can be found in S7 Data. DOY, day of year; OTU, operational taxonomic unit.
(PDF)

S9 Fig Phenotypic (NMDS2) values for the outlier SNP, Chr02N_57831909. The x-axis shows jittered genotypic value, with 0 and 2 as homozygotes, and 1 as the heterozygote. Points are colored by population. Subpopulations with the _admix suffix show substantial admixture from other populations. Data underlying this figure can be found in S8 Data. NMDS, nonmetric multidimensional scaling.
(PDF)

S10 Fig Sequencing coverage and structural variation in the region of interest. The upper panel shows Loess-smoothed normalized sequencing coverage in this region for 3 representative genotypes. Colors indicate genotype, with the blue line showing an individual homozygous for the major allele, and red and green showing a heterozygote and the minor allele homozygote, respectively. The lower panel shows structural variation in the same region. Insertions and deletions in the region of our outlier SNP were overrepresented in the area surrounding the candidate genes, indicating structural variation across genotypes that may account for genetic and phenotypic differences. Red diamonds show deletions, and blue triangles show insertions. Red arrows with text show nearby genes. Data underlying this figure can be found in S9 Data. SNP, single nucleotide polymorphism.
(PDF)

S1 Table Core microbiome taxonomy and functional guilds. This table can be found as a spreadsheet in S10 Data.
(PDF)

S2 Table Bootstrap *p*-value for temporal network distance tests in MINA. This table can be found as a spreadsheet in S11 Data.
(PDF)

S3 Table Sample information. Ecotype and subpopulation membership are estimated using SNP data. Latitude and longitude denote original collection site, if known. This table can be found as a spreadsheet in S12 Data. SNP, single nucleotide polymorphism.
(PDF)

S4 Table PCR conditions for ITS amplification. This table can be found as a spreadsheet in S13 Data.
(PDF)

S5 Table RNA sequencing transcripts for 3 crRLK genes. This table can be found as a spreadsheet in S14 Data.
(PDF)

S6 Table Fungal contaminants removed by the decontam package. This table can be found as a spreadsheet in S15 Data.
(PDF)

S1 Data NMDS and PCoA results for KBS site. Custom R scripts to create trajectory plots. KBS, Kellogg Biological Station; NMDS, nonmetric multidimensional scaling.
(ZIP)

S2 Data Site comparison NMDS results. NMDS, nonmetric multidimensional scaling.
(ZIP)

S3 Data Genetic and microbiome distance data. (ZIP)

S4 Data NMDS values used for GWAS. RNA sequencing FPKM values. FPKM, fragments per kilobase of transcript per million mapped reads; GWAS, genome-wide association study; NMDS, nonmetric multidimensional scaling.
(ZIP)

S5 Data Network diagram data. Raw tables for network statistics.
(ZIP)

S6 Data Confirmatory samples NMDS and PERMANOVA results. NMDS, nonmetric multidimensional scaling; PERMANOVA, permutational multivariate analysis of variance.
(ZIP)

S7 Data Class-level comparisons between OTUs. OTU, operational taxonomic unit.
(ZIP)

S8 Data Allele distribution at the outlier SNP Chr02N_57831909. SNP, single nucleotide polymorphism.
(ZIP)

S9 Data Sequencing coverage and insertion/deletion locations within the outlier region on Chr02N. (ZIP)

S10 Data Core OTUs taxonomic information and citations for guild estimates. OTU, operational taxonomic unit.
(ZIP)

S11 Data MINA results. (ZIP)

S12 Data Collection site information for switchgrass genotypes. (ZIP)

S13 Data PCR conditions. (ZIP)

S14 Data Raw transcript counts for RNA sequencing. (ZIP)

S15 Data Taxonomic information for contaminants removed from analyses. (ZIP)

## Abbreviations:

crRLK: cysteine-rich receptor-like kinase
DOY: day of year
FDR: false discovery rate
GWA: genome-wide association
GWAS: genome-wide association study
HSD: honest significant difference
ITS: internal transcribed spacer
KBS: Kellogg Biological Station
LSU: large subunit
MAF: minor allele frequency
MSU: Michigan State University
MTV-LMM: microbial temporal variability mixed linear model
NMDS: nonmetric multidimensional scaling; OTU, operational taxonomic unit; PERMANOVA, permutational multivariate analysis of variance
SNP: single nucleotide polymorphism
SVD: singular value decomposition
