# Supplementary material for: Host genotype controls ecological change in the leaf fungal microbiome
Source: PLoS Biol. 2022 Aug 11;20(8):e3001681. doi: 10.1371/journal.pbio.3001681 (PMC9371330; doi:10.1371/journal.pbio.3001681)
Supplement: S1 Fig — Data underlying this figure can be found in S1 Data. KBS, Kellogg Biological Station; NMDS, nonmetric multidimensional scaling. (PDF) [file pbio.3001681.s001.pdf]

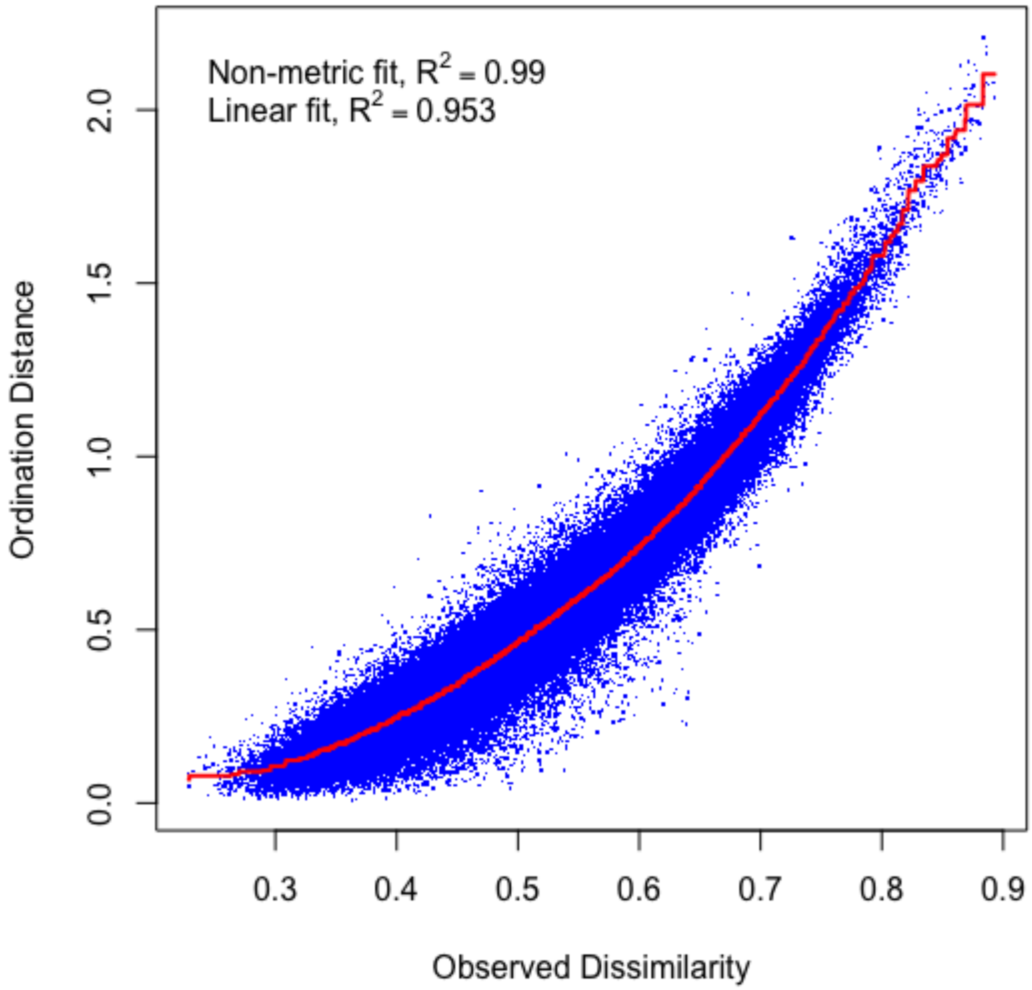

Figure S1: Shepard stress plot for NMDS of Kellogg Biological Station site. Data underlying this figure can be found in FigS1 Data.
