# Supplementary material for: Host genotype controls ecological change in the leaf fungal microbiome
Source: PLoS Biol. 2022 Aug 11;20(8):e3001681. doi: 10.1371/journal.pbio.3001681 (PMC9371330; doi:10.1371/journal.pbio.3001681)
Supplement: S2 Fig — Latitude and longitude are available in S3 Table. The base map uses points from the US Census Bureau, implemented in the maps R package (https://www.census.gov/geographies/mapping-files/time-series/geo/carto-boundary-file.html). Data underlying this figure can be found in S3 Table. (PDF) [file pbio.3001681.s002.pdf]

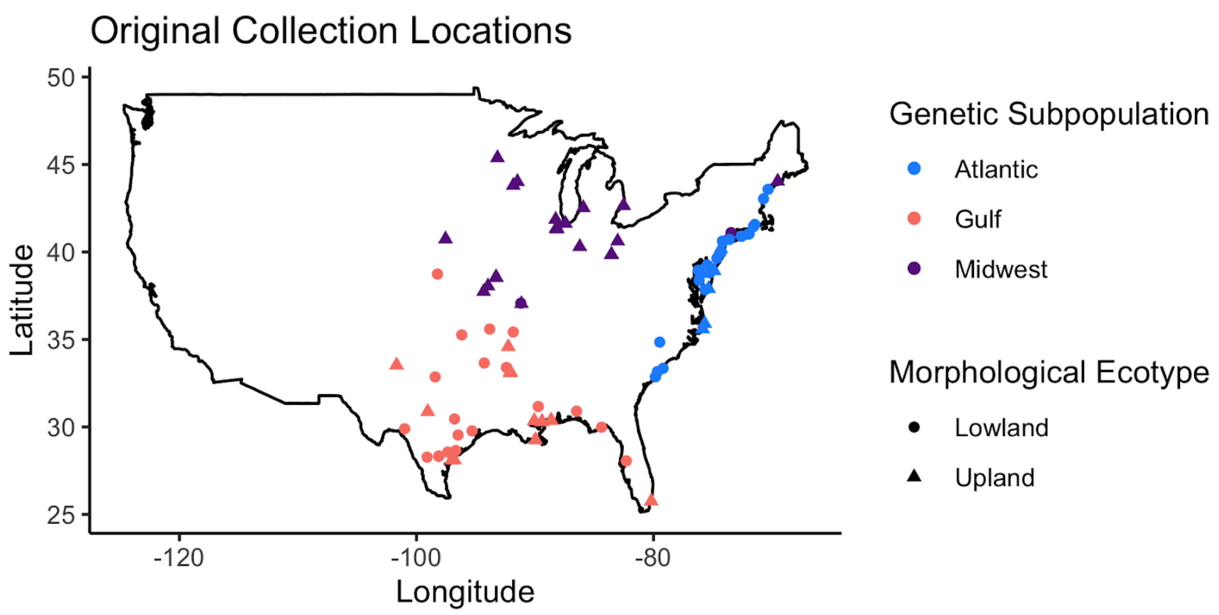

Figure S2: Original collection locations for samples. Latitude and Longitude are available in Table S3. The base map uses points from the US Census Bureau, implemented in the maps R package (https://www.census.gov/geographies/mapping-files/time-series/geo/carto-boundary-file.html). Data underlying this figure can be found in Table S3.
