# Supplementary material for: Host genotype controls ecological change in the leaf fungal microbiome
Source: PLoS Biol. 2022 Aug 11;20(8):e3001681. doi: 10.1371/journal.pbio.3001681 (PMC9371330; doi:10.1371/journal.pbio.3001681)
Supplement: S3 Fig — Each icon represents one sample taken. We sampled 106 genotypes at 5 time points at the focal site in Hickory Corners, Michigan, and 8 genotypes at the 3 other sites. We sampled roughly equal numbers of each subpopulation throughout. Sites are shown from northern (KBS, Michigan) to southern (Kingsville, Texas). KBS, Kellogg Biological Station. (PDF) [file pbio.3001681.s003.pdf]

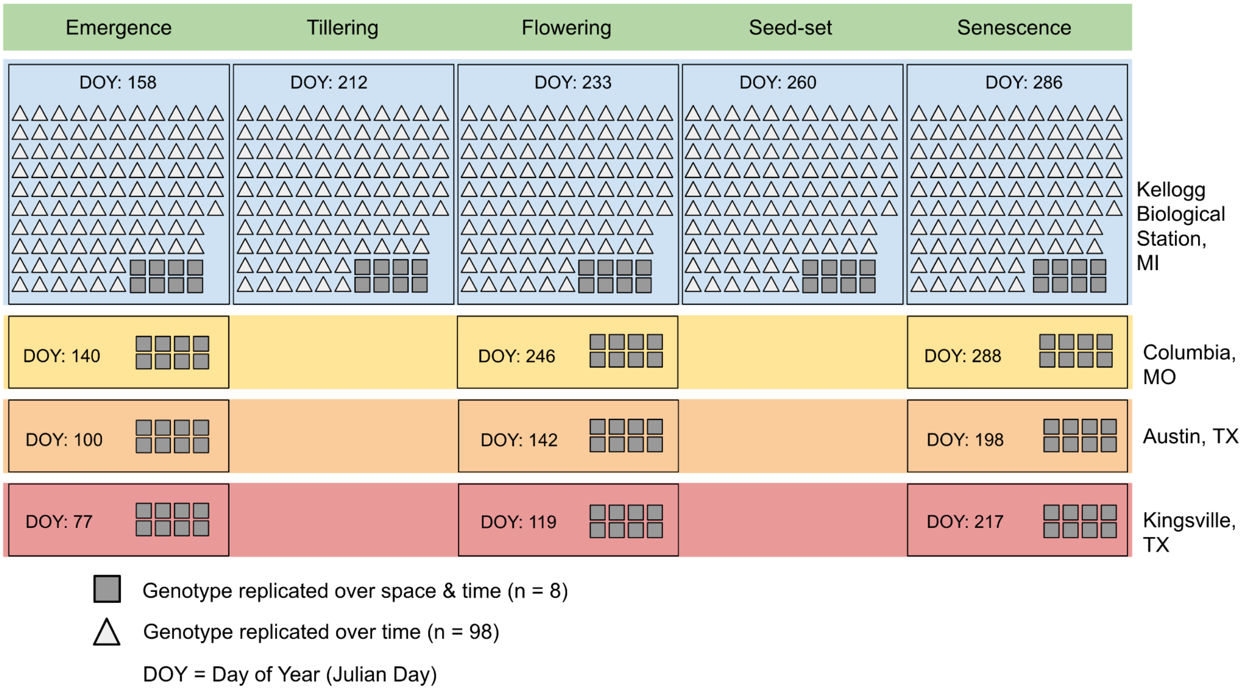

Figure S3. Sampling scheme.
Each icon represents one sample taken. We sampled 106 genotypes at five time points at the focal site in Hickory Corners, MI, and 8 genotypes at the three other sites. We sampled roughly equal numbers of each subpopulation throughout. Sites are shown from northern (Kellogg Biological Station, MI) to southern (Kingsville, TX).
