# Supplementary material for: Host genotype controls ecological change in the leaf fungal microbiome
Source: PLoS Biol. 2022 Aug 11;20(8):e3001681. doi: 10.1371/journal.pbio.3001681 (PMC9371330; doi:10.1371/journal.pbio.3001681)
Supplement: S5 Fig — A. DOY158; B. DOY212; C. DOY233; D. DOY286. Data underlying this figure can be found in S4 Data. (PDF) [file pbio.3001681.s005.pdf]

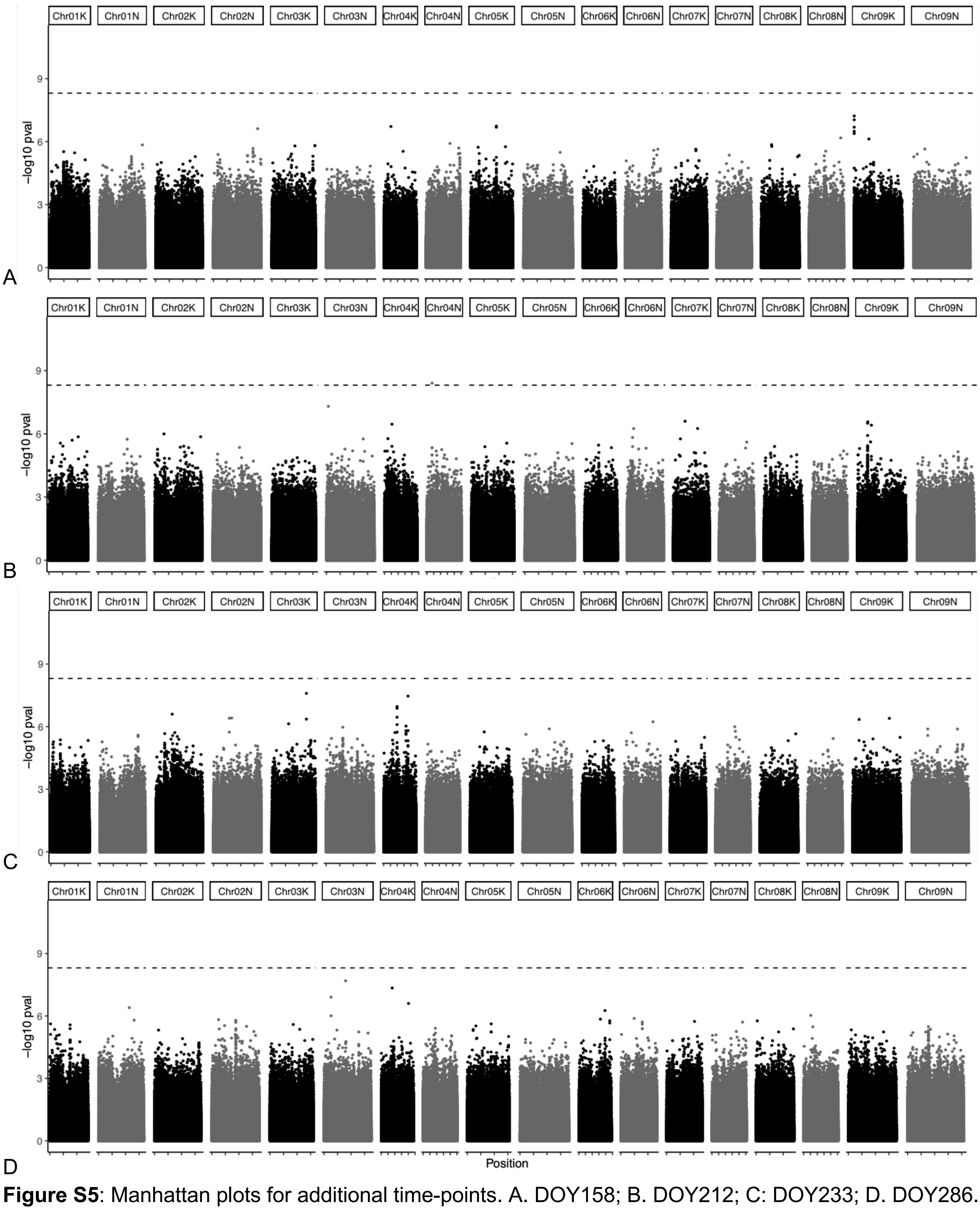

Figure S5: Manhattan plots for additional time-points. A. DOY158; B. DOY212; C: DOY233; D. DOY286. Data underlying this figure can be found in Fig4 Data.
