# Supplementary material for: Host genotype controls ecological change in the leaf fungal microbiome
Source: PLoS Biol. 2022 Aug 11;20(8):e3001681. doi: 10.1371/journal.pbio.3001681 (PMC9371330; doi:10.1371/journal.pbio.3001681)
Supplement: S6 Fig — Data underlying this figure can be found in S4 Data. GWAS, genome-wide association study. (PDF) [file pbio.3001681.s006.pdf]

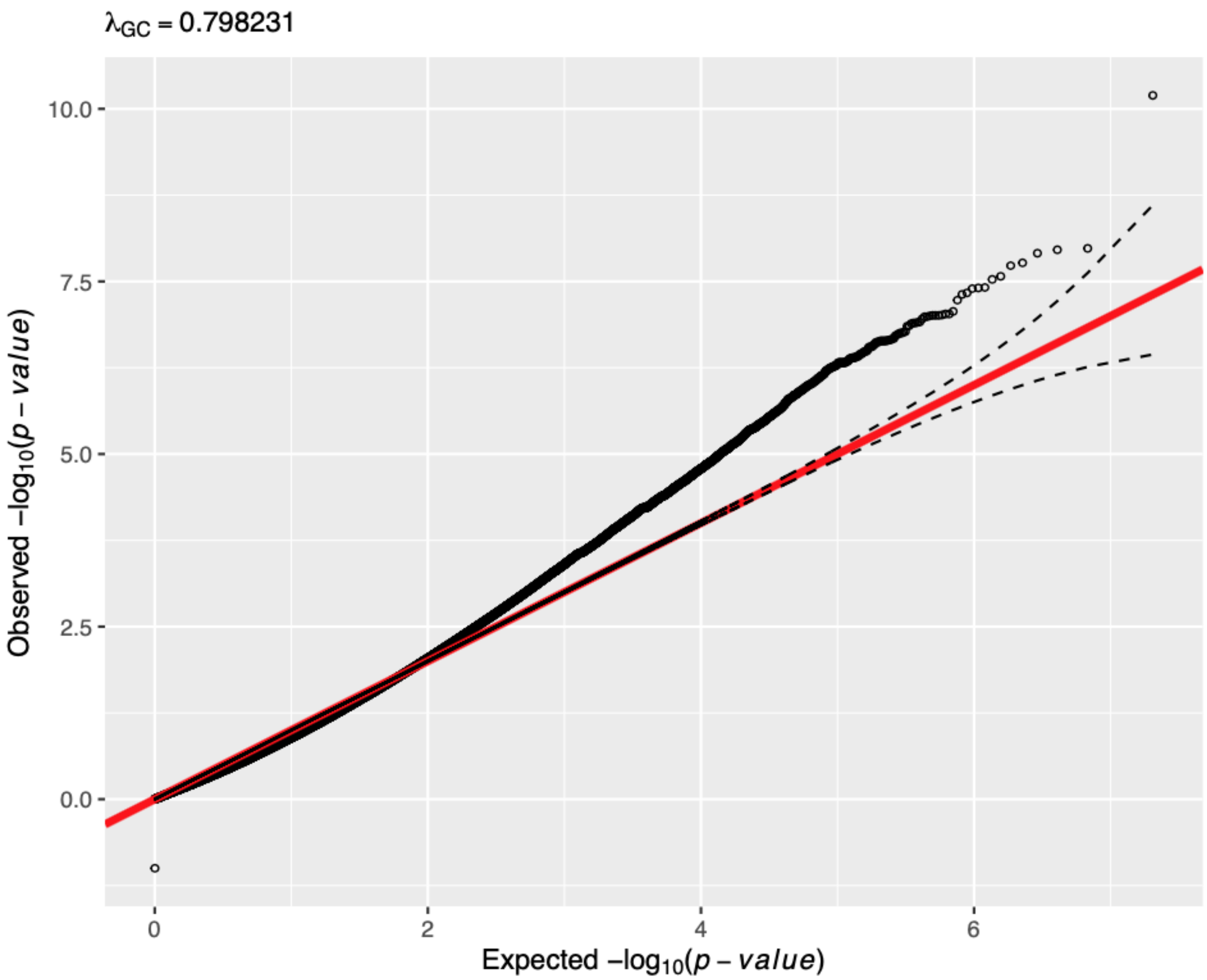

Figure S6: Quantile-Quantile plot for Microbiome GWAS results showing an excess of observed low p-values. Data underlying this figure can be found in Fig4 Data.
