# Supplementary material for: Host genotype controls ecological change in the leaf fungal microbiome
Source: PLoS Biol. 2022 Aug 11;20(8):e3001681. doi: 10.1371/journal.pbio.3001681 (PMC9371330; doi:10.1371/journal.pbio.3001681)
Supplement: S7 Fig — These results confirm that the locus has a significant impact on the fungal community. The figures at left show NMDS plots of the fungal community at 2 time points sampled in 2021. The right tables show PERMANOVA results for these samples, indicating a significant effect of allele on community structure. Data underlying this figure can be found in S6 Data. NMDS, nonmetric multidimensional scaling; PERMANOVA, permutational multivariate analysis of variance. (PDF) [file pbio.3001681.s007.pdf]

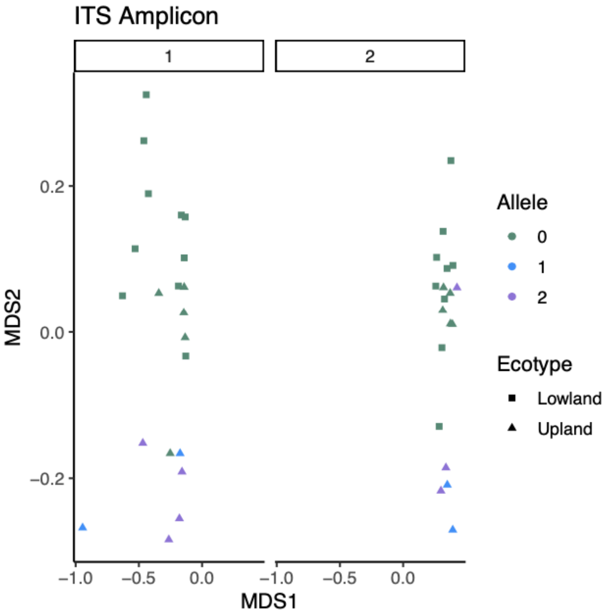

ITS PERMANOVA

	Df	SS	Rsq	F	Pr(>F)
Allele	1	0.214	0.044	2.586241	0.019
Date	1	1.488	0.308	17.996376	0.001
PC1	1	0.101	0.021	1.224042	0.185
PC2	1	0.132	0.027	1.599140	0.110
Residual	35	2.895	0.599		
Total	39	4.831	1.000		

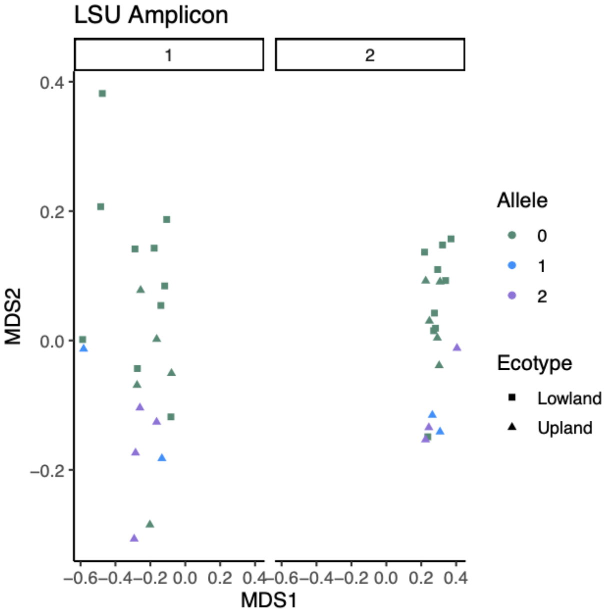

LSU PERMANOVA

	Df	SS	Rsq	F	Pr(>F)
allele	1	0.267	0.056	3.401969	0.002
numdate	1	1.453	0.306	18.507556	0.001
PC1	1	0.143	0.030	1.822064	0.055
PC2	1	0.142	0.030	1.812353	0.053
Residual	35	2.749	0.578		
Total	39	4.755	1.000		

Figure S7: Fungal community differences across alternate allele states at locus Chr02N_57831909. These results confirm that the locus has a significant impact on the fungal community. The figures at left show NMDS plots of the fungal community at two time points sampled in 2021. The right tables show PERMANOVA results for these samples, indicating a significant effect of allele on community structure. Data underlying this figure can be found in FigS7 Data.
