# Supplementary material for: Host genotype controls ecological change in the leaf fungal microbiome
Source: PLoS Biol. 2022 Aug 11;20(8):e3001681. doi: 10.1371/journal.pbio.3001681 (PMC9371330; doi:10.1371/journal.pbio.3001681)
Supplement: S8 Fig — Column facets show DOY, and row facets show whether connections were negative or positive. Data underlying this figure can be found in S7 Data. DOY, day of year; OTU, operational taxonomic unit. (PDF) [file pbio.3001681.s008.pdf]

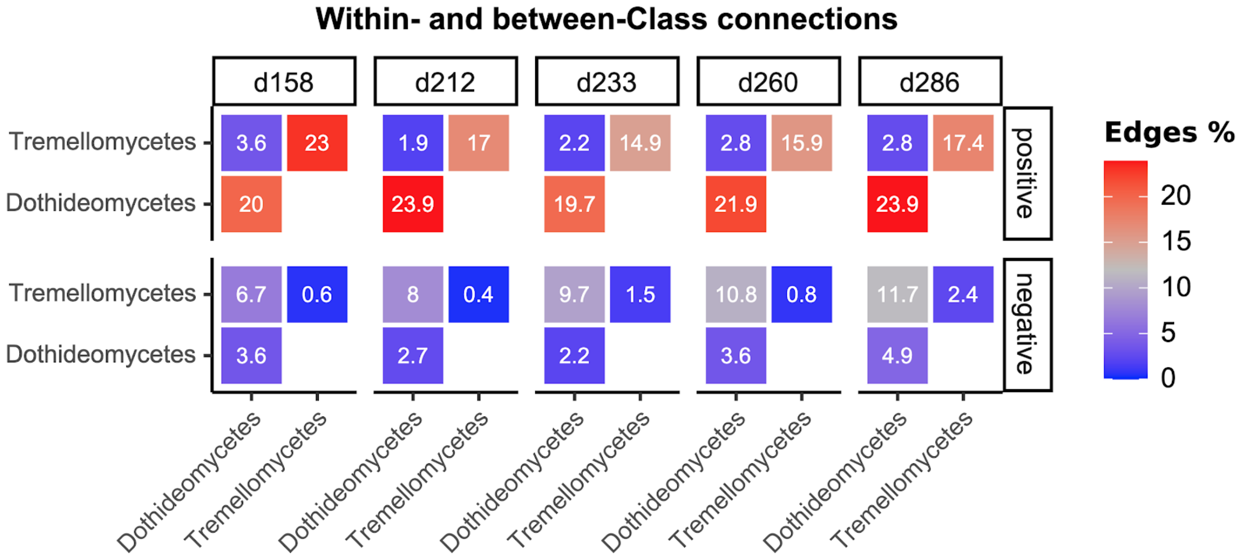

Figure S8: Class-level comparison of the proportion of edges linking OTUs within or between each Class in a time point. Column facets show day of year (DOY), and row facets show whether connections were negative or positive. Data underlying this figure can be found in FigS8 Data.
