# Supplementary material for: Host genotype controls ecological change in the leaf fungal microbiome
Source: PLoS Biol. 2022 Aug 11;20(8):e3001681. doi: 10.1371/journal.pbio.3001681 (PMC9371330; doi:10.1371/journal.pbio.3001681)
Supplement: S9 Fig — The x-axis shows jittered genotypic value, with 0 and 2 as homozygotes, and 1 as the heterozygote. Points are colored by population. Subpopulations with the _admix suffix show substantial admixture from other populations. Data underlying this figure can be found in S8 Data. NMDS, nonmetric multidimensional scaling. (PDF) [file pbio.3001681.s009.pdf]

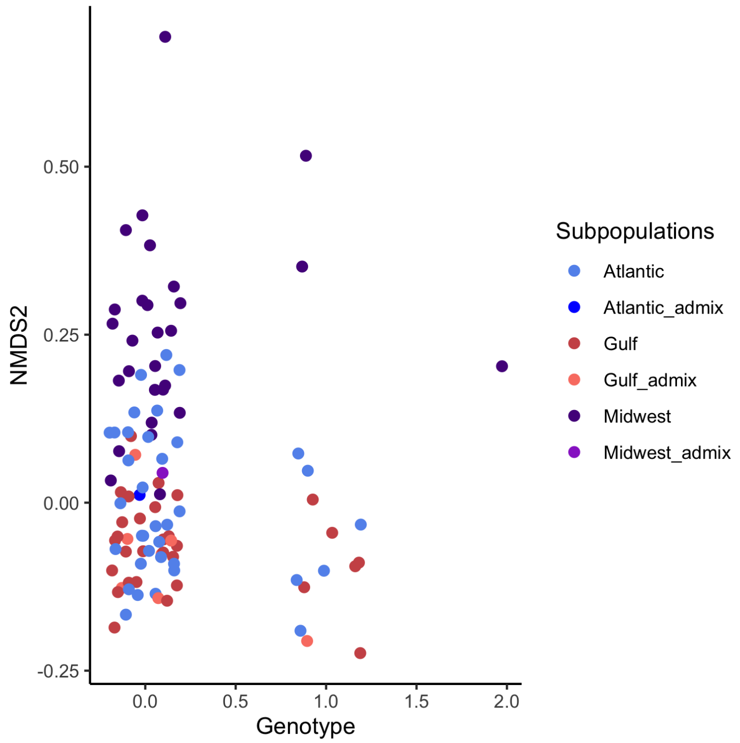

Figure S9: Phenotypic (NMDS2) values for the outlier SNP, Chr02N_57831909. The x-axis shows jittered genotypic value, with 0 and 2 as homozygotes, and 1 as the heterozygote. Points are colored by population. Subpopulations with the _admix suffix show substantial admixture from other populations. Data underlying this figure can be found in FigS9 Data.
