# Supplementary material for: Host genotype controls ecological change in the leaf fungal microbiome
Source: PLoS Biol. 2022 Aug 11;20(8):e3001681. doi: 10.1371/journal.pbio.3001681 (PMC9371330; doi:10.1371/journal.pbio.3001681)
Supplement: S10 Fig — The upper panel shows Loess-smoothed normalized sequencing coverage in this region for 3 representative genotypes. Colors indicate genotype, with the blue line showing an individual homozygous for the major allele, and red and green showing a heterozygote and the minor allele homozygote, respectively. The lower panel shows structural variation in the same region. Insertions and deletions in the region of our outlier SNP were overrepresented in the area surrounding the candidate genes, indicating structural variation across genotypes that may account for genetic and phenotypic differences. Red diamonds show deletions, and blue triangles show insertions. Red arrows with text show nearby genes. Data underlying this figure can be found in S9 Data. SNP, single nucleotide polymorphism. (PDF) [file pbio.3001681.s010.pdf]

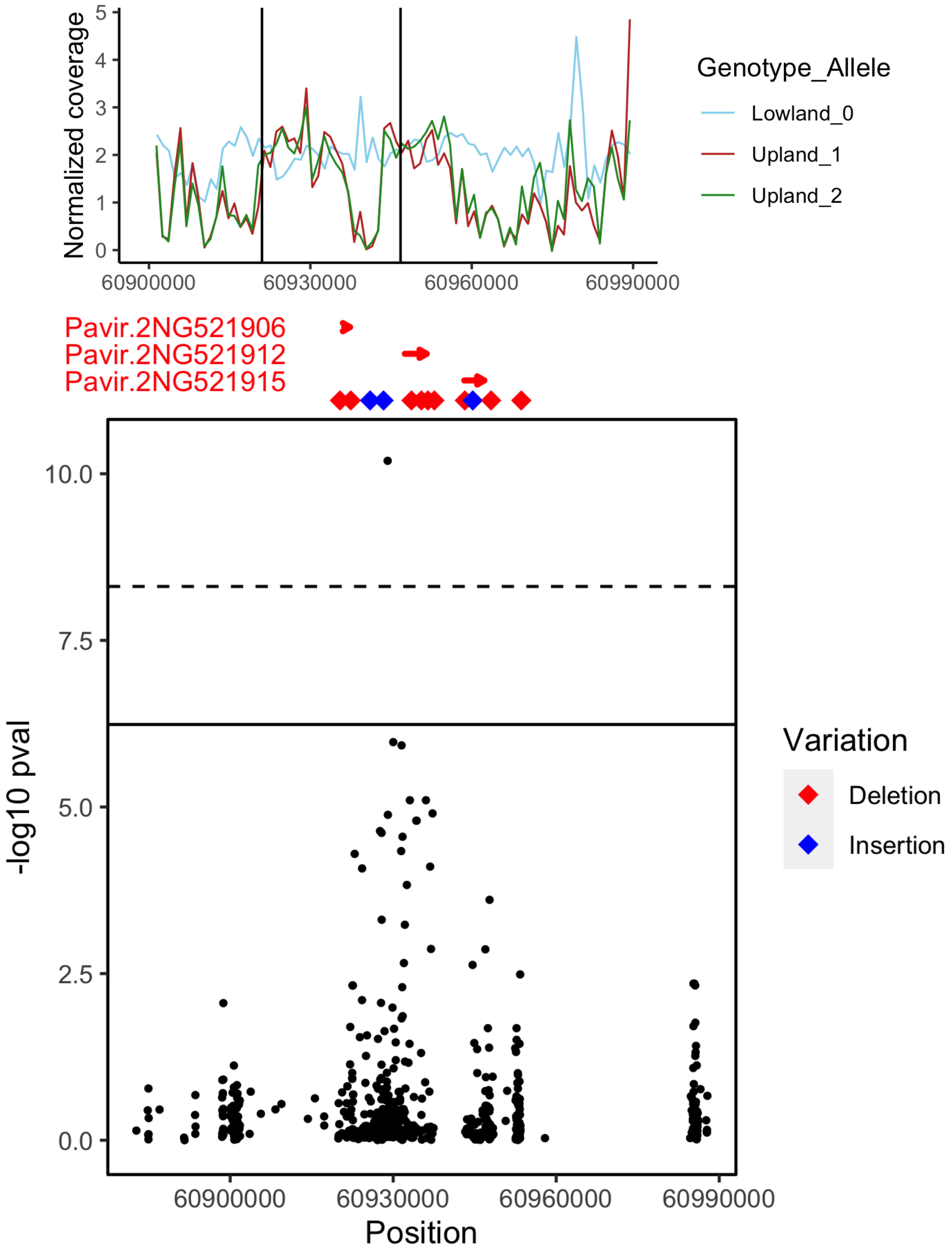

Figure S10: Sequencing coverage and structural variation in the region of interest. The upper panel shows Loess-smoothed normalized sequencing coverage in this region for three representative genotypes. Colors indicate genotype, with the blue line showing an individual homozygous for the major allele, and red and green showing a heterozygote and the minor allele homozygote, respectively. The lower panel shows structural variation in the same region. Insertions and deletions in the region of our outlier SNP were overrepresented in the area surrounding the candidate genes, indicating structural variation across genotypes that may account for genetic and phenotypic differences. Red diamonds show deletions, and blue triangles show insertions. Red arrows with text show nearby genes. Data underlying this figure can be found in FigS10 Data.
