# Supplementary material for: Host genotype controls ecological change in the leaf fungal microbiome
Source: PLoS Biol. 2022 Aug 11;20(8):e3001681. doi: 10.1371/journal.pbio.3001681 (PMC9371330; doi:10.1371/journal.pbio.3001681)
Supplement: S1 Table — This table can be found as a spreadsheet in S10 Data. (PDF) [file pbio.3001681.s011.pdf]

Table S1: Core microbiome taxonomy and functional guilds. This table can be found as a spreadsheet in TableS1 Data.

OTU_ID	Phylum	Class	Genus	Species	BLAST Percent ID	Functional Guild	Guild Source
OTU_2	Ascomycota	Dothideomycetes	Alternaria	NA	100	Pathogen	Source
OTU_32	Ascomycota	Dothideomycetes	Alternaria	NA	100	Pathogen	Source
OTU_6104	Ascomycota	Dothideomycetes	Aureobasidium	NA	96.094	Yeast or Yeast-like	Source
OTU_1760	Basidiomycota	Tremellomycetes	Bulleromyces	Bulleromyces albus	0	Yeast or Yeast-like	Source
OTU_5936	Basidiomycota	Tremellomycetes	Bulleromyces	Bulleromyces albus	0	Yeast or Yeast-like	Source
OTU_738	Basidiomycota	Tremellomycetes	Bulleromyces	Bulleromyces albus	99.422	Yeast or Yeast-like	Source
OTU_392	Basidiomycota	Tremellomycetes	Bulleromyces	Bulleromyces albus	0	Yeast or Yeast-like	Source
OTU_47	Ascomycota	Dothideomycetes	Cladosporium	Cladosporium grevilleae	100	saprobe	Source
OTU_21	Ascomycota	Dothideomycetes	Coniothyrium	Coniothyrium	100	Other Pathogen	Source
OTU_380	Basidiomycota	Tremellomycetes	Cryptococcus	NA	100	Yeast or Yeast-like	Source
OTU_2137	Ascomycota	Dothideomycetes	Didymella	NA	99.471	Other Pathogen	Source
OTU_877	Basidiomycota	Tremellomycetes	Dioszegia	Dioszegia athyri	98.919	Yeast or Yeast-like	Source
OTU_1303	Basidiomycota	Tremellomycetes	Dioszegia	Dioszegia athyri	98.413	Yeast or Yeast-like	Source
OTU_1615	Basidiomycota	Tremellomycetes	Dioszegia	Dioszegia athyri	98.919	Yeast or Yeast-like	Source
OTU_464	Basidiomycota	Tremellomycetes	Dioszegia	Dioszegia hungarica	0	Yeast or Yeast-like	Source
OTU_1011	Basidiomycota	Tremellomycetes	Dioszegia	Dioszegia hungarica	98.817	Yeast or Yeast-like	Source
OTU_5498	Basidiomycota	Tremellomycetes	Dioszegia	Dioszegia hungarica	0	Yeast or Yeast-like	Source
OTU_822	Basidiomycota	Tremellomycetes	Dioszegia	NA	100	Yeast or Yeast-like	Source
OTU_1367	Basidiomycota	Tremellomycetes	Dioszegia	NA	100	Yeast or Yeast-like	Source
OTU_414	Basidiomycota	Tremellomycetes	Dioszegia	NA	100	Yeast or Yeast-like	Source
OTU_2213	Basidiomycota	Tremellomycetes	Dioszegia	NA	100	Yeast or Yeast-like	Source
OTU_677	Ascomycota	Dothideomycetes	Dissoconium	Dissoconium eucalypti	99	Other Pathogen	Source
OTU_18	Ascomycota	Dothideomycetes	Dissoconium	NA	98.02	Other Pathogen	
OTU_35	Ascomycota	Dothideomycetes	Epicoccum	Epicoccum dendrobii	100	Mycoparasite	Source
OTU_165	Ascomycota	Dothideomycetes	Epicoccum	Epicoccum dendrobii	97.98	Mycoparasite	Source
OTU_2231	Basidiomycota	Cystobasidiomycetes	Erythrobasidium	Erythrobasidium hasegawianum	97.959	Yeast or Yeast-like	Source
OTU_770	Basidiomycota	Cystobasidiomycetes	Erythrobasidium	NA	98.469	Yeast or Yeast-like	Source

OTU_52	Basidiomycota	Cystobasidiomycetes	Erythrobasidium	Erythrobasidium yunnanense	98.985	Yeast or Yeast-like	Source
OTU_33	Basidiomycota	Tremellomycetes	Filobasidium	Filobasidium floriforme	99	Yeast or Yeast-like	Source
OTU_980	Basidiomycota	Tremellomycetes	Filobasidium	NA	0	Yeast or Yeast-like	Source
OTU_1226	Basidiomycota	Tremellomycetes	Filobasidium	Filobasidium wieringae	100	Yeast or Yeast-like	Source
OTU_37	Ascomycota	Sordariomycetes	Fusarium	Fusarium sporotrichioides	100	Pathogen	Source
OTU_2922	Basidiomycota	Tremellomycetes	Hannaella	Hannaella sinensis	0	Yeast or Yeast-like	Source
OTU_791	Basidiomycota	Tremellomycetes	Hannaella	Hannaella sinensis	0	Yeast or Yeast-like	Source
OTU_1724	Basidiomycota	Tremellomycetes	Hannaella	Hannaella sinensis	0	Yeast or Yeast-like	Source
OTU_14	Ascomycota	Dothideomycetes	Keissleriella	Keissleriella caraganae	100	Other Pathogen	Source
OTU_92	Basidiomycota	Agaricostilbomycetes	Kondoa	Kondoa	97.5	Yeast or Yeast-like	Source
OTU_24	Basidiomycota	Agaricostilbomycetes	Kondoa	Kondoa	99	Yeast or Yeast-like	Source
OTU_38	Basidiomycota	Agaricostilbomycetes	Kondoa	Kondoa miscanthi	99.502	Yeast or Yeast-like	Source
OTU_28	Basidiomycota	Agaricostilbomycetes	Kondoa	NA	94.634	Yeast or Yeast-like	Source
OTU_72	Ascomycota	Dothideomycetes	Leptospora	Leptospora	99		
OTU_6	Ascomycota	Sordariomycetes	Microdochium	Microdochium seminicola	95.05	Pathogen	Source
OTU_158	Ascomycota	Dothideomycetes	Mycosphaerella	Mycosphaerella tassiana	99.5	Pathogen	Source
OTU_7	Ascomycota	NA	NA	NA	0		
OTU_426	Ascomycota	Dothideomycetes	NA	NA	98.942		
OTU_1076	Ascomycota	Dothideomycetes	NA	NA	99.474		
OTU_1690	Ascomycota	Dothideomycetes	NA	NA	98.454		
OTU_42	Ascomycota	Dothideomycetes	NA	NA	0		
OTU_5	Ascomycota	Dothideomycetes	NA	NA	100		
OTU_103	NA	NA	NA	NA	100		
OTU_115	NA	NA	NA	NA	99.5		
OTU_15	Ascomycota	Leotiomycetes	NA	NA	89.447		
OTU_93	Basidiomycota	Microbotryomycetes	NA	NA	0		
OTU_56	Ascomycota	Sordariomycetes	NA	NA	100		
OTU_341	Ascomycota	Dothideomycetes	NA	NA	98.947		
OTU_1207	Ascomycota	Dothideomycetes	NA	NA	98.469		
OTU_1723	Ascomycota	Dothideomycetes	NA	NA	99.479		
OTU_610	Ascomycota	Dothideomycetes	NA	NA	99.471		

OTU_20	Ascomycota	Dothideomycetes	NA	NA	100		
OTU_79	Ascomycota	Dothideomycetes	NA	NA	99.5		
OTU_705	Ascomycota	Dothideomycetes	NA	NA	98.953		
OTU_2655	Ascomycota	Dothideomycetes	NA	NA	100		
OTU_1041	Ascomycota	Dothideomycetes	NA	NA	97		
OTU_11	Ascomycota	Dothideomycetes	NA	NA	100		
OTU_8	Ascomycota	Dothideomycetes	NA	NA	100		
OTU_13	Ascomycota	Dothideomycetes	NA	NA	100		
OTU_5444	Ascomycota	Dothideomycetes	NA	NA	97.015		
OTU_23	Ascomycota	Leotiomycetes	NA	NA	100		
OTU_628	Ascomycota	Leotiomycetes	NA	NA	96.5		
OTU_248	Basidiomycota	Tremellomycetes	NA	NA	94.767		
OTU_505	Basidiomycota	Tremellomycetes	NA	NA	94.35		
OTU_1234	Basidiomycota	Tremellomycetes	NA	NA	94.767		
OTU_3488	Basidiomycota	Tremellomycetes	NA	NA	93.605		
OTU_4633	Basidiomycota	Tremellomycetes	NA	NA	94.186		
OTU_6081	Basidiomycota	Tremellomycetes	NA	NA	94.767		
OTU_5180	Basidiomycota	Tremellomycetes	NA	NA	94.767		
OTU_41	Basidiomycota	Tremellomycetes	NA	NA	95.567		
OTU_3292	Ascomycota	Dothideomycetes	Neoascochyta	Neoascochyta exitialis	99.487	Pathogen	Source
OTU_443	Ascomycota	Dothideomycetes	Neoascochyta	NA	98.953	Pathogen	Source
OTU_429	Ascomycota	Dothideomycetes	Neoascochyta	NA	100	Pathogen	Source
OTU_340	Ascomycota	Dothideomycetes	Neodevriesia	Neodevriesia poagena	100		Source
OTU_61	Ascomycota	Sordariomycetes	Nigrospora	Nigrospora oryzae	100	Pathogen	Source
OTU_669	Basidiomycota	Tremellomycetes	Papiliotrema	Papiliotrema pseudoalba	0	Yeast or Yeast-like	Source
OTU_6048	Basidiomycota	Tremellomycetes	Papiliotrema	Papiliotrema pseudoalba	0	Yeast or Yeast-like	Source
OTU_1993	Basidiomycota	Tremellomycetes	Papiliotrema	NA	100	Yeast or Yeast-like	Source
OTU_394	Basidiomycota	Tremellomycetes	Papiliotrema	NA	100	Yeast or Yeast-like	Source
OTU_7324	Basidiomycota	Tremellomycetes	Papiliotrema	NA	99.425	Yeast or Yeast-like	Source
OTU_6867	Basidiomycota	Tremellomycetes	Papiliotrema	NA	100	Yeast or Yeast-like	Source
OTU_4109	Basidiomycota	Tremellomycetes	Papiliotrema	NA	98.851	Yeast or Yeast-like	Source

OTU_5837	Basidiomycota	Tremellomycetes	Papiliotrema	NA	98.851	Yeast or Yeast-like	Source
OTU_4177	Basidiomycota	Tremellomycetes	Papiliotrema	NA	98.857	Yeast or Yeast-like	Source
OTU_74	Ascomycota	Dothideomycetes	Paraophiobolus	Paraophiobolus arundinis	100		Source
OTU_49	Ascomycota	Dothideomycetes	Paraphaeosphaeria	Paraphaeosphaeria michotii	100		Source
OTU_137	Basidiomycota	Agaricomycetes	Peniophora	NA	99		Source
OTU_43	Ascomycota	Dothideomycetes	Phaeosphaeria	Phaeosphaeria	99.497	Pathogen	Source
OTU_125	Ascomycota	Dothideomycetes	Phaeosphaeria	Phaeosphaeria	100	Pathogen	
OTU_83	Ascomycota	Dothideomycetes	Phaeosphaeria	NA	92.04	Pathogen	
OTU_232	Ascomycota	Dothideomycetes	Phaeosphaeria	NA	95.855		
OTU_3	Ascomycota	Dothideomycetes	Phoma	NA	100		Source
OTU_59	Ascomycota	Dothideomycetes	Ramularia	NA	0		Source
OTU_1684	Basidiomycota	Tremellomycetes	Saitozyma	Saitozyma paraflava	0		Source
OTU_1028	Basidiomycota	Tremellomycetes	Saitozyma	Saitozyma paraflava	0		Source
OTU_50	Ascomycota	Sordariomycetes	Sarocladium	NA	100		Source
OTU_91	Ascomycota	Dothideomycetes	Septoria	NA	100		Source
OTU_143	Ascomycota	Dothideomycetes	Setomelanomma	Setomelanomma	0		Source
OTU_4	Ascomycota	Dothideomycetes	Sphaerellopsis	Sphaerellopsis filum	97	Mycoparasite	Source
OTU_31	Basidiomycota	Microbotryomycetes	Sporobolomyces	Sporobolomyces patagonicus	98.985	Yeast or Yeast-like	Source
OTU_17	Basidiomycota	Microbotryomycetes	Sporobolomyces	Sporobolomyces phaffii	100	Yeast or Yeast-like	Source
OTU_12	Basidiomycota	Microbotryomycetes	Sporobolomyces	Sporobolomyces roseus	99.5		Source
OTU_30	Ascomycota	Dothideomycetes	Stagonospora	Stagonospora pseudovitensis	98		Source
OTU_71	Basidiomycota	Cystobasidiomycetes	Symmetrospora	Symmetrospora coprosmae	100		Source
OTU_476	Basidiomycota	Cystobasidiomycetes	Symmetrospora	Symmetrospora gracilis	100		Source
OTU_86	Basidiomycota	Cystobasidiomycetes	Symmetrospora	NA	99.479		Source
OTU_1663	Basidiomycota	Cystobasidiomycetes	Symmetrospora	NA	99.49		Source
OTU_22	Ascomycota	Taphrinomycetes	Taphrina	Taphrina	100	Pathogen	Source
OTU_64	Ascomycota	Taphrinomycetes	Taphrina	Taphrina communis	100	Pathogen	Source
OTU_16	Ascomycota	Taphrinomycetes	Taphrina	Taphrina confusa	99	Pathogen	Source
OTU_26	Ascomycota	Taphrinomycetes	Taphrina	Taphrina letifera	99.5	Pathogen	Source
OTU_10	Ascomycota	Taphrinomycetes	Taphrina	NA	87.624	Pathogen	Source
OTU_54	Ascomycota	Taphrinomycetes	Taphrina	Taphrina tormentillae	99.5	Pathogen	Source

OTU_105	Basidiomycota	Exobasidiomycetes	Tilletiopsis	Tilletiopsis washingtonensis	98	Source
OTU_75	Basidiomycota	Tremellomycetes	Udeniomyces	Udeniomyces pyricola	100	Source
OTU_9	Ascomycota	Dothideomycetes	Uwebraunia	Uwebraunia dekkeri	100	Source
OTU_4086	Ascomycota	Dothideomycetes	Vagicola	Vagicola chlamydospora	96.535	Source
OTU_2473	Basidiomycota	Tremellomycetes	Vishniacozyma	Vishniacozyma victoriae	96.196	Source
OTU_1571	Basidiomycota	Tremellomycetes	Vishniacozyma	Vishniacozyma victoriae	98.37	Source
OTU_3311	Basidiomycota	Tremellomycetes	Vishniacozyma	Vishniacozyma victoriae	100	Source
OTU_979	Ascomycota	Dothideomycetes	Zymoseptoria	NA	96.891	Source
