# Supplementary material for: Host genotype controls ecological change in the leaf fungal microbiome
Source: PLoS Biol. 2022 Aug 11;20(8):e3001681. doi: 10.1371/journal.pbio.3001681 (PMC9371330; doi:10.1371/journal.pbio.3001681)
Supplement: S2 Table — This table can be found as a spreadsheet in S11 Data. (PDF) [file pbio.3001681.s012.pdf]

Table S2: Bootstrap p-values for temporal network distance tests in MINA. This table can be found as a spreadsheet in TableS2 Data.

	DOY 158	DOY 212	DOY 233	DOY 260	DOY 286
**DOY 158**	0.741	0.001	0.001	0.001	0.001
**DOY 212**		0.431	0.025	0.001	0.001
**DOY 233**			0.574	0.015	0.001
**DOY 260**				0.588	0.025
**DOY 286**					0.578
