# Supplementary material for: Host genotype controls ecological change in the leaf fungal microbiome
Source: PLoS Biol. 2022 Aug 11;20(8):e3001681. doi: 10.1371/journal.pbio.3001681 (PMC9371330; doi:10.1371/journal.pbio.3001681)
Supplement: S3 Table — Ecotype and subpopulation membership are estimated using SNP data. Latitude and longitude denote original collection site, if known. This table can be found as a spreadsheet in S12 Data. SNP, single nucleotide polymorphism. (PDF) [file pbio.3001681.s013.pdf]

Table S3: Sample information. Ecotype and subpopulation membership are estimated using SNP data. Latitude and longitude denote original collection site, if known. This table can be found as a spreadsheet in TableS3 Data.

PLOT_ID	PLANT_ID	Ecotype_SNP	Subpopulation	Latitude	Longitude
M1001	J208.B	Lowland	eastcoast_admixed	34.845	-79.470
M1005	J297.A	Lowland	Texas	NA	NA
M1010	J427.B	Lowland	eastcoast	40.620	-74.180
M1107	J585.A	Lowland	eastcoast	32.865	-79.837
M1116	J294.A	Upland	Texas_admixed	28.110	-97.029
M1120	J368.B	Upland	midwest	42.528	-85.922
M1202	J344.C	Upland	midwest	41.305	-88.172
M1209	J330.A	Lowland	Texas	28.276	-99.101
M1218	J499.A	Upland	Gulfcoast	30.348	-90.057
M1401	J022.B	Lowland	Texas	28.333	-98.118
M1407	J186.A	Lowland	Gulfcoast_admixed	29.986	-84.387
M1408	J491.B	Upland	midwest	38.050	-93.967
M1411	J661.A	Lowland	eastcoast	37.841	-75.654
M1417	J589.B	Upland	eastcoast	37.907	-75.351
M1420	J314.A	Lowland	Texas_admixed	29.775	-95.310
M1602	J483.C	Upland	Gulfcoast	33.085	-92.070
M1605	J448.A	Upland	midwest	NA	NA
M1801	J028.C	Lowland	Texas	35.593	-93.825
M1804	J461.C	Upland	Gulfcoast	30.299	-89.407
M1806	J496.A	Upland	Gulfcoast	29.262	-89.952
M2002	J462.C	NA	NA	30.481	-92.668
M2005	J306.A	Upland	Texas	30.874	-99.050
M2015	J433.B	Lowland	eastcoast	41.020	-72.010
M2018	J614.B	Upland	eastcoast	38.935	-74.906
M2020	J482.B	Upland	Gulfcoast	33.150	-92.076
M2108	J502.C	Upland	Gulfcoast	30.377	-88.634
M2201	J538.C	Lowland	eastcoast	41.017	-72.001
M2214	J305.A	Upland	Texas_admixed	28.117	-96.800
M2216	J396.A	Upland	midwest	43.800	-91.830
M2217	J355.A	Upland	midwest	41.347	-88.139
M2218	J022.C	Lowland	Texas	28.333	-98.118
M2401	J672.A	Lowland	eastcoast	39.255	-75.466
M2402	J598.B	Upland	eastcoast	38.772	-75.977
M2417	J348.C	Upland	midwest	40.620	-83.021
M2601	J481.A	Lowland	Gulfcoast_admixed	33.397	-92.414
M2607	J536.C	Lowland	eastcoast	41.104	-73.451

M2614	J222.A	Lowland	Texas	30.460	-96.785
M2620	J682.C	Upland	eastcoast	40.179	-74.317
M2802	J315.A	Lowland	Texas	NA	NA
M2806	BLK.17	NA	NA	NA	NA
M2920	J331.A	Lowland	Texas	29.538	-96.493
M3001	J299.A	Lowland	Texas_admixed	NA	NA
M3003	J663.B	Lowland	eastcoast	38.374	-76.149
M3007	BLK.20	NA	NA	NA	NA
M3012	J502.C	Upland	Gulfcoast	30.377	-88.634
M3201	J308.A	Lowland	Texas_admixed	28.567	-97.352
M3202	J534.C	Lowland	eastcoast	40.586	-74.119
M3205	J580.A	Upland	midwest	41.861	-88.254
M3209	BLK.21	NA	NA	NA	NA
M3210	J657.C	Lowland	midwest	37.073	-91.197
M3220	J582.C	Upland	midwest	41.365	-88.187
M3520	J461.B	Upland	Gulfcoast	30.299	-89.407
M3606	J245.A	Lowland	Texas	32.860	-98.420
M3613	J065.A	Lowland	Texas	28.060	-82.300
M3615	J651.C	Lowland	Texas	NA	NA
M3618	J635.A	Lowland	eastcoast	43.580	-70.329
M3803	J353.B	Upland	midwest	39.854	-83.531
M3819	J318.A	Lowland	Texas	28.654	-96.681
M4001	J484.B	Lowland	Texas	33.658	-94.281
M4017	J379.B	Upland	midwest	42.648	-82.529
M4021	J673.D	Lowland	eastcoast	38.820	-75.228
M4209	J390.B	Upland	midwest	40.300	-86.220
M4210	J016.C	Lowland	Texas	35.260	-96.180
M4211	J018.C	Lowland	Texas	35.427	-91.837
M4410	J504.C	Lowland	Gulfcoast	31.170	-89.730
M4412	J024.A	Upland	midwest	51.219	4.402
M4416	J004.B	Lowland	Texas	38.737	-98.228
M4418	J355.C	Upland	midwest	41.347	-88.139
M4419	J416.B	Lowland	eastcoast	40.600	-74.130
M4520	J499.C	Upland	Gulfcoast	30.348	-90.057
M4602	J656.B	Upland	midwest	37.044	-91.141
M4610	J536.A	Lowland	midwest_admixed	41.104	-73.451
M4617	J482.A	Upland	Gulfcoast	33.150	-92.076
M4802	J660.C	Lowland	Texas	29.898	-100.997
M4805	J538.A	Lowland	eastcoast	41.017	-72.001

M4817	J614.A	Upland	eastcoast	38.935	-74.906
M5401	J341.A	Upland	Texas	33.533	-101.680
M5404	J352.B	Upland	midwest	39.839	-83.573
M5405	J045.C	Upland	midwest	40.740	-97.546
M5409	J650.A	Upland	Texas_admixed	25.762	-80.192
M5413	J646.C	Lowland	eastcoast	41.463	-71.554
M5418	J489.A	Upland	midwest	37.737	-94.328
M5804	J419.A	Lowland	Texas	NA	NA
M5809	J387.C	Upland	midwest	38.551	-93.299
M5810	J477.C	Upland	Gulfcoast_admixed	34.587	-92.254
M5811	BLK.50	NA	NA	NA	NA
M5812	J386.C	Upland	midwest	38.548	-93.258
M5813	J596.C	Lowland	eastcoast	38.952	-76.234
M5817	J258.A	Upland	midwest	45.382	-93.164
M6005	J636.C	Upland	midwest	44.043	-69.516
M6012	J593.B	Upland	eastcoast	35.600	-75.848
M6016	J522.B	Lowland	eastcoast	39.644	-74.644
M6019	J613.C	Upland	eastcoast	35.908	-75.676
M6204	J521.A	Lowland	eastcoast	39.965	-74.312
M6215	J602.C	Lowland	eastcoast	33.352	-79.194
M6221	J344.A	Upland	midwest	41.305	-88.172
M6607	J646.A	Lowland	eastcoast	41.463	-71.554
M6608	J607.A	Lowland	eastcoast	41.575	-71.454
M6610	J521.C	Lowland	eastcoast	39.965	-74.312
M6611	J609.C	Lowland	eastcoast	33.167	-79.666
M6613	J432.B	Lowland	eastcoast	41.040	-71.930
M6614	J385.A	Upland	midwest	38.532	-93.291
M6615	J634.A	Lowland	eastcoast	43.038	-70.716
M6705	J646.B	Lowland	eastcoast	41.463	-71.554
M6706	J428.B	Lowland	eastcoast	40.720	-73.580
M6708	J521.B	Lowland	eastcoast	39.965	-74.312
M6711	J403.B	Upland	midwest	44.020	-91.480
M6712	J525.C	Lowland	eastcoast	40.903	-72.571
M6713	J525.A	Lowland	eastcoast	40.903	-72.571
M6714	J456.A	Lowland	Gulfcoast	30.900	-86.494
M6802	J525.B	Lowland	eastcoast	40.903	-72.571
M6803	J379.A	Upland	midwest	42.648	-82.529
M6813	J393.B	Upland	midwest	41.630	-87.430
M6817	J538.B	Lowland	eastcoast	41.017	-72.001
