# Supplementary material for: Host genotype controls ecological change in the leaf fungal microbiome
Source: PLoS Biol. 2022 Aug 11;20(8):e3001681. doi: 10.1371/journal.pbio.3001681 (PMC9371330; doi:10.1371/journal.pbio.3001681)
Supplement: S4 Table — This table can be found as a spreadsheet in S13 Data. (PDF) [file pbio.3001681.s014.pdf]

Table S4: PCR conditions for ITS amplification. This table can be found as a spreadsheet in TableS4 Data.

Round 1 (10X)		Round 2 (10X)		Round 3 (15X)	
**Temp (°C)**	**Time**	**Temp (°C)**	**Time**	**Temp (°C)**	**Time**
94°	5:00	94°	5:00	94°	5:00
94°	0:30	94°	0:30	94°	0:30
**50°**	0:30	**50°**	0:35	**63°**	0:35
72°	1:20	72°	1:20	72°	1:20
72°	7:00	72°	7:00	72°	7:00
4°	inf	4°	inf	4°	inf

round 1----- 1x plate			
**Reagents**	**µL**	**Reactions**	**Total (µL)**
Dream Taq GREEN	10.4	104	1081.6
ITS1F	0.625	104	65
ITS2	0.625	104	65
BSA 3%	3.35	104	348.4
H2O	1	104	104
**Total (µL)**	**16**		**1,664**
DNA (µL)	4		
Final reaction volume	20		
**round 2-----1x plate**			
Dream Taq GREEN	**µL**	**Reactions**	**Total (µL)**
master MIX	6.25	104	650
ITS1F*	0.375	104	39
ITS2*	0.375	104	39
BSA 3%	2	104	208
H2O	2	104	208
**Total (µL)**	**11**		**1,144**
DNA (µL)	2		
Final reaction volume	13		
**round 3 ----- 1x plate**			
Dream Taq GREEN	**µL**	**Reactions**	**Total (µL)**
master MIX	8	104	832
Forward primer F	0.5	104	52
Barcode primers R	-	-	-
BSA 3%	1.5	104	156
H2O	0	104	0
**Total (µL)**	**10+1ulBar**		**1,040**
DNA (µL)	4		
