# Supplementary material for: Host genotype controls ecological change in the leaf fungal microbiome
Source: PLoS Biol. 2022 Aug 11;20(8):e3001681. doi: 10.1371/journal.pbio.3001681 (PMC9371330; doi:10.1371/journal.pbio.3001681)
Supplement: S5 Table — This table can be found as a spreadsheet in S14 Data. (PDF) [file pbio.3001681.s015.pdf]

Table S5: RNA-sequencing transcripts for three crRLK genes. This table can be found as a spreadsheet in TableS5 Data.

Genotype	Transcript_Count	Gene	Site
AP13	0	Pavir.2NG521906	Columbia
AP13	1	Pavir.2NG521906	Columbia
AP13	0	Pavir.2NG521906	Columbia
AP13	3	Pavir.2NG521906	Columbia
AP13	0	Pavir.2NG521906	Columbia
DAC	60	Pavir.2NG521906	Columbia
DAC	78	Pavir.2NG521906	Columbia
DAC	99	Pavir.2NG521906	Columbia
DAC	113	Pavir.2NG521906	Columbia
VS16	0	Pavir.2NG521906	Columbia
VS16	0	Pavir.2NG521906	Columbia
VS16	0	Pavir.2NG521906	Columbia
VS16	0	Pavir.2NG521906	Columbia
WBC	0	Pavir.2NG521906	Columbia
WBC	0	Pavir.2NG521906	Columbia
WBC	0	Pavir.2NG521906	Columbia
WBC	1	Pavir.2NG521906	Columbia
WBC	0	Pavir.2NG521906	Columbia
AP13	1	Pavir.2NG521906	KBS
AP13	2	Pavir.2NG521906	KBS
AP13	1	Pavir.2NG521906	KBS
AP13	2	Pavir.2NG521906	KBS
AP13	1	Pavir.2NG521906	KBS
DAC	41	Pavir.2NG521906	KBS
DAC	15	Pavir.2NG521906	KBS
DAC	11	Pavir.2NG521906	KBS
VS16	0	Pavir.2NG521906	KBS
VS16	1	Pavir.2NG521906	KBS
VS16	0	Pavir.2NG521906	KBS
VS16	2	Pavir.2NG521906	KBS
VS16	0	Pavir.2NG521906	KBS
WBC	0	Pavir.2NG521906	KBS
WBC	0	Pavir.2NG521906	KBS
WBC	1	Pavir.2NG521906	KBS
WBC	1	Pavir.2NG521906	KBS
WBC	1	Pavir.2NG521906	KBS
AP13	1	Pavir.2NG521906	Austin
AP13	2	Pavir.2NG521906	Austin
AP13	0	Pavir.2NG521906	Austin

AP13	1	Pavir.2NG521906	Austin
AP13	0	Pavir.2NG521906	Austin
DAC	79	Pavir.2NG521906	Austin
DAC	0	Pavir.2NG521906	Austin
DAC	76	Pavir.2NG521906	Austin
VS16	0	Pavir.2NG521906	Austin
VS16	0	Pavir.2NG521906	Austin
VS16	0	Pavir.2NG521906	Austin
VS16	0	Pavir.2NG521906	Austin
VS16	0	Pavir.2NG521906	Austin
WBC	0	Pavir.2NG521906	Austin
WBC	1	Pavir.2NG521906	Austin
WBC	2	Pavir.2NG521906	Austin
WBC	4	Pavir.2NG521906	Austin
WBC	4	Pavir.2NG521906	Austin
AP13	123	Pavir.2NG521912	Columbia
AP13	153	Pavir.2NG521912	Columbia
AP13	101	Pavir.2NG521912	Columbia
AP13	139	Pavir.2NG521912	Columbia
AP13	102	Pavir.2NG521912	Columbia
DAC	520	Pavir.2NG521912	Columbia
DAC	699	Pavir.2NG521912	Columbia
DAC	361	Pavir.2NG521912	Columbia
DAC	684	Pavir.2NG521912	Columbia
VS16	613	Pavir.2NG521912	Columbia
VS16	523	Pavir.2NG521912	Columbia
VS16	711	Pavir.2NG521912	Columbia
VS16	548	Pavir.2NG521912	Columbia
WBC	149	Pavir.2NG521912	Columbia
WBC	110	Pavir.2NG521912	Columbia
WBC	110	Pavir.2NG521912	Columbia
WBC	186	Pavir.2NG521912	Columbia
WBC	199	Pavir.2NG521912	Columbia
AP13	209	Pavir.2NG521912	KBS
AP13	148	Pavir.2NG521912	KBS
AP13	69	Pavir.2NG521912	KBS
AP13	140	Pavir.2NG521912	KBS
AP13	189	Pavir.2NG521912	KBS
DAC	382	Pavir.2NG521912	KBS
DAC	107	Pavir.2NG521912	KBS
DAC	64	Pavir.2NG521912	KBS

VS16	495	Pavir.2NG521912	KBS
VS16	552	Pavir.2NG521912	KBS
VS16	256	Pavir.2NG521912	KBS
VS16	64	Pavir.2NG521912	KBS
VS16	989	Pavir.2NG521912	KBS
WBC	194	Pavir.2NG521912	KBS
WBC	104	Pavir.2NG521912	KBS
WBC	235	Pavir.2NG521912	KBS
WBC	55	Pavir.2NG521912	KBS
WBC	102	Pavir.2NG521912	KBS
AP13	132	Pavir.2NG521912	Austin
AP13	171	Pavir.2NG521912	Austin
AP13	166	Pavir.2NG521912	Austin
AP13	66	Pavir.2NG521912	Austin
AP13	125	Pavir.2NG521912	Austin
DAC	532	Pavir.2NG521912	Austin
DAC	555	Pavir.2NG521912	Austin
DAC	526	Pavir.2NG521912	Austin
VS16	448	Pavir.2NG521912	Austin
VS16	589	Pavir.2NG521912	Austin
VS16	981	Pavir.2NG521912	Austin
VS16	514	Pavir.2NG521912	Austin
VS16	977	Pavir.2NG521912	Austin
WBC	166	Pavir.2NG521912	Austin
WBC	128	Pavir.2NG521912	Austin
WBC	115	Pavir.2NG521912	Austin
WBC	473	Pavir.2NG521912	Austin
WBC	256	Pavir.2NG521912	Austin
AP13	8	Pavir.2NG521915	Columbia
AP13	8	Pavir.2NG521915	Columbia
AP13	6	Pavir.2NG521915	Columbia
AP13	5	Pavir.2NG521915	Columbia
AP13	7	Pavir.2NG521915	Columbia
DAC	70	Pavir.2NG521915	Columbia
DAC	76	Pavir.2NG521915	Columbia
DAC	84	Pavir.2NG521915	Columbia
DAC	224	Pavir.2NG521915	Columbia
VS16	323	Pavir.2NG521915	Columbia
VS16	254	Pavir.2NG521915	Columbia
VS16	231	Pavir.2NG521915	Columbia
VS16	275	Pavir.2NG521915	Columbia

WBC	4	Pavir.2NG521915	Columbia
WBC	1	Pavir.2NG521915	Columbia
WBC	0	Pavir.2NG521915	Columbia
WBC	12	Pavir.2NG521915	Columbia
WBC	0	Pavir.2NG521915	Columbia
AP13	47	Pavir.2NG521915	KBS
AP13	19	Pavir.2NG521915	KBS
AP13	15	Pavir.2NG521915	KBS
AP13	24	Pavir.2NG521915	KBS
AP13	23	Pavir.2NG521915	KBS
DAC	287	Pavir.2NG521915	KBS
DAC	239	Pavir.2NG521915	KBS
DAC	378	Pavir.2NG521915	KBS
VS16	918	Pavir.2NG521915	KBS
VS16	471	Pavir.2NG521915	KBS
VS16	470	Pavir.2NG521915	KBS
VS16	437	Pavir.2NG521915	KBS
VS16	586	Pavir.2NG521915	KBS
WBC	35	Pavir.2NG521915	KBS
WBC	8	Pavir.2NG521915	KBS
WBC	29	Pavir.2NG521915	KBS
WBC	6	Pavir.2NG521915	KBS
WBC	9	Pavir.2NG521915	KBS
AP13	2	Pavir.2NG521915	Austin
AP13	10	Pavir.2NG521915	Austin
AP13	10	Pavir.2NG521915	Austin
AP13	5	Pavir.2NG521915	Austin
AP13	18	Pavir.2NG521915	Austin
DAC	368	Pavir.2NG521915	Austin
DAC	404	Pavir.2NG521915	Austin
DAC	134	Pavir.2NG521915	Austin
VS16	1141	Pavir.2NG521915	Austin
VS16	322	Pavir.2NG521915	Austin
VS16	272	Pavir.2NG521915	Austin
VS16	280	Pavir.2NG521915	Austin
VS16	425	Pavir.2NG521915	Austin
WBC	4	Pavir.2NG521915	Austin
WBC	4	Pavir.2NG521915	Austin
WBC	10	Pavir.2NG521915	Austin
WBC	5	Pavir.2NG521915	Austin
WBC	3	Pavir.2NG521915	Austin
