# Supplementary material for: Host genotype controls ecological change in the leaf fungal microbiome
Source: PLoS Biol. 2022 Aug 11;20(8):e3001681. doi: 10.1371/journal.pbio.3001681 (PMC9371330; doi:10.1371/journal.pbio.3001681)
Supplement: S6 Table — This table can be found as a spreadsheet in S15 Data. (PDF) [file pbio.3001681.s016.pdf]

Table S6: Fungal contaminants removed by the decontam package. This table can be found as a spreadsheet in TableS6 Data.

Contaminant Fungi

	OTU_ID	Phylum	Species	BestMatch	ID_confidence	Abundance
OTU_7689	OTU_7689	Ascomycota	Cyberlindnera jadinii	Cyberlindnera jadinii	98.958	0.0013297
OTU_129	OTU_129	Basidiomycota	Rhodotorula diobovata	Rhodotorula diobovata	98.500	0.0012824
OTU_1184	OTU_1184	Ascomycota	NA	Penicillium sp.	100.000	0.0010461
OTU_3664	OTU_3664	Ascomycota	NA	Pleosporales	99.497	0.0009121
OTU_1269	OTU_1269	Ascomycota	NA	Pleosporales	100.000	0.0008759
OTU_4014	OTU_4014	Ascomycota	NA	Saccharomycetales	100.000	0.0006480
OTU_803	OTU_803	Ascomycota	NA	Ascomycota	100.000	0.0006408
OTU_639	OTU_639	Basidiomycota	NA	Clavaria sp.	98.995	0.0005878
OTU_1448	OTU_1448	Basidiomycota	NA	Agaricomycetes	79.612	0.0005852
OTU_2561	OTU_2561	Ascomycota	NA	Lasiosphaeriaceae	99.497	0.0005655
OTU_566	OTU_566	Ascomycota	Chaetomium	Chaetomium	99.000	0.0005546
OTU_6377	OTU_6377	Ascomycota	Lasiodiplodia bruguierae	Lasiodiplodia bruguierae	100.000	0.0004852
OTU_4736	OTU_4736	Basidiomycota	NA	Trechisporales	99.500	0.0003888
OTU_933	OTU_933	Basidiomycota	Tausonia pullulans	Tausonia pullulans	100.000	0.0003788
OTU_4596	OTU_4596	Ascomycota	NA	Magnaporthaceae	0.000	0.0003713
OTU_2185	OTU_2185	Ascomycota	NA	Ascomycota	80.220	0.0003311
OTU_1335	OTU_1335	Mortierellomycota	Mortierella alpina	Mortierella alpina	100.000	0.0003127
OTU_1420	OTU_1420	NA	NA	Fungi	99.500	0.0002847
OTU_828	OTU_828	Ascomycota	NA	Talaromyces sp.	99.500	0.0002441
OTU_1591	OTU_1591	Ascomycota	Trichocladium opacum	Trichocladium opacum	99.500	0.0002166
OTU_6937	OTU_6937	Glomeromycota	NA	Glomeraceae	0.000	0.0002145
OTU_1653	OTU_1653	Ascomycota	NA	Incertae sedis	100.000	0.0001704
OTU_2333	OTU_2333	Ascomycota	NA	Saccharomycetales	0.000	0.0001526
OTU_1964	OTU_1964	Ascomycota	Comoclathris	Comoclathris	99.500	0.0001152
OTU_7842	OTU_7842	Basidiomycota	Malassezia globosa	Malassezia globosa	95.025	0.0001140
OTU_1162	OTU_1162	Ascomycota	Toxicocladosporium irritans	Toxicocladosporium irritans	99.005	0.0001045
OTU_4812	OTU_4812	Ascomycota	NA	Eurotiales	100.000	0.0001037
OTU_1380	OTU_1380	Ascomycota	Plenodomus biglobosus	Plenodomus biglobosus	100.000	0.0000898
OTU_4566	OTU_4566	Ascomycota	NA	Saccharomycetales	0.000	0.0000765
OTU_234	OTU_234	Ascomycota	NA	Stachybotryaceae	98.010	0.0000581
OTU_2171	OTU_2171	Ascomycota	NA	Helotiaceae	99.000	0.0000551
OTU_351	OTU_351	Ascomycota	NA	Dothideomycetes	95.918	0.0000428
OTU_2350	OTU_2350	Basidiomycota	Malassezia restricta	Malassezia restricta	100.000	0.0000427
OTU_3690	OTU_3690	Chytridiomycota	NA	Chytridiomycota	84.878	0.0000373
OTU_314	OTU_314	Ascomycota	NA	Ascomycota	0.000	0.0000353
OTU_696	OTU_696	Ascomycota	NA	Dinemasporium sp.	97.462	0.0000277
OTU_3044	OTU_3044	Mortierellomycota	NA	Mortierella sp.	99.492	0.0000274
OTU_2983	OTU_2983	Ascomycota	NA	Helotiales	98.507	0.0000133
OTU_2805	OTU_2805	Ascomycota	NA	Pseudogymnoascus sp.	100.000	0.0000109
OTU_2881	OTU_2881	Ascomycota	NA	Arthrinium sp.	99.500	0.0000106
OTU_6435	OTU_6435	Basidiomycota	Malassezia globosa	Malassezia globosa	100.000	0.0000094
OTU_2384	OTU_2384	Basidiomycota	NA	Delicatula sp.	97.512	0.0000093
OTU_5725	OTU_5725	Mortierellomycota	Mortierella gamsii	Mortierella gamsii	99.492	0.0000091
OTU_7956	OTU_7956	Chytridiomycota	NA	Chytridiomycota	97.487	0.0000090
OTU_792	OTU_792	Ascomycota	NA	Ascomycota	0.000	0.0000075
OTU_3757	OTU_3757	Mortierellomycota	Mortierella exigua	Mortierella exigua	98.010	0.0000057
OTU_4057	OTU_4057	Ascomycota	NA	Hypocreales	96.500	0.0000050
OTU_5812	OTU_5812	Mortierellomycota	Mortierella	Mortierella	100.000	0.0000049
OTU_7623	OTU_7623	Basidiomycota	NA	Agaricales	97.500	0.0000047
OTU_1512	OTU_1512	Mortierellomycota	NA	Mortierella sp.	100.000	0.0000042
OTU_5299	OTU_5299	Ascomycota	NA	Helotiales	97.970	0.0000030
OTU_1694	OTU_1694	Ascomycota	Trichoderma atroviride	Trichoderma atroviride	100.000	0.0000029
OTU_1667	OTU_1667	Ascomycota	NA	Paraophiobolus sp.	94.500	0.0000023
OTU_3601	OTU_3601	Basidiomycota	Clitopilus prunulus	Clitopilus prunulus	100.000	0.0000021
OTU_6360	OTU_6360	Basidiomycota	NA	Agaricomycetes	96.000	0.0000019
OTU_6694	OTU_6694	Basidiomycota	Vishniacozyma dimennae	Vishniacozyma dimennae	0.000	0.0000019
OTU_2574	OTU_2574	Ascomycota	NA	Pleosporales	88.384	0.0000017
OTU_1947	OTU_1947	Ascomycota	NA	Pleosporales	100.000	0.0000016
OTU_6251	OTU_6251	Ascomycota	NA	Hypocreales	91.489	0.0000008
OTU_3169	OTU_3169	Mortierellomycota	Mortierella alpina	Mortierella alpina	100.000	0.0000006
OTU_3823	OTU_3823	NA	NA	Fungi	0.000	0.0000006
OTU_2395	OTU_2395	NA	NA	Fungi	0.000	0.0000004
OTU_3649	OTU_3649	Ascomycota	NA	Ascomycota	0.000	0.0000003
OTU_5151	OTU_5151	Chytridiomycota	Spizellomyces plurigibbosus	Spizellomyces plurigibbosus	100.000	0.0000003
OTU_7142	OTU_7142	Ascomycota	NA	Sordariales	97.990	0.0000003
OTU_3816	OTU_3816	Ascomycota	NA	Pleosporales	96.020	0.0000002
OTU_7754	OTU_7754	Ascomycota	NA	Pleosporales	95.500	0.0000002
OTU_4399	OTU_4399	Ascomycota	NA	Humicola sp.	100.000	0.0000002
OTU_5332	OTU_5332	Ascomycota	Ascobolus	Ascobolus	98.500	0.0000002
OTU_4208	OTU_4208	Ascomycota	NA	Lasiosphaeriaceae	99.497	0.0000002
OTU_4504	OTU_4504	Basidiomycota	Lepista sordida	Lepista sordida	99.000	0.0000002
OTU_7612	OTU_7612	Ascomycota	NA	Stagonospora sp.	89.394	0.0000001
OTU_7216	OTU_7216	Basidiomycota	NA	Malasseziales	99.500	0.0000001
OTU_7882	OTU_7882	Chytridiomycota	NA	Spizellomyces sp.	0.000	0.0000001

Contaminant Fungi

	OTU_ID	Phylum	Species	BestMatch	ID_confidence	Abundance
OTU_7689	OTU_7689	Ascomycota	Cyberlindnera jadinii	Cyberlindnera jadinii	98.958	0.0013297
OTU_129	OTU_129	Basidiomycota	Rhodotorula diobovata	Rhodotorula diobovata	98.500	0.0012824
OTU_1184	OTU_1184	Ascomycota	NA	Penicillium sp.	100.000	0.0010461
OTU_3664	OTU_3664	Ascomycota	NA	Pleosporales	99.497	0.0009121
OTU_1269	OTU_1269	Ascomycota	NA	Pleosporales	100.000	0.0008759
OTU_4014	OTU_4014	Ascomycota	NA	Saccharomycetales	100.000	0.0006480
OTU_803	OTU_803	Ascomycota	NA	Ascomycota	100.000	0.0006408
OTU_639	OTU_639	Basidiomycota	NA	Clavaria sp.	98.995	0.0005878
OTU_1448	OTU_1448	Basidiomycota	NA	Agaricomycetes	79.612	0.0005852
OTU_2561	OTU_2561	Ascomycota	NA	Lasiosphaeriaceae	99.497	0.0005655
OTU_566	OTU_566	Ascomycota	Chaetomium	Chaetomium	99.000	0.0005546
OTU_6377	OTU_6377	Ascomycota	Lasiodiplodia bruguierae	Lasiodiplodia bruguierae	100.000	0.0004852
OTU_4736	OTU_4736	Basidiomycota	NA	Trechisporales	99.500	0.0003888
OTU_933	OTU_933	Basidiomycota	Tausonia pullulans	Tausonia pullulans	100.000	0.0003788
OTU_4596	OTU_4596	Ascomycota	NA	Magnaporthaceae	0.000	0.0003713
OTU_2185	OTU_2185	Ascomycota	NA	Ascomycota	80.220	0.0003311
OTU_1335	OTU_1335	Mortierellomycota	Mortierella alpina	Mortierella alpina	100.000	0.0003127
OTU_1420	OTU_1420	NA	NA	Fungi	99.500	0.0002847
OTU_828	OTU_828	Ascomycota	NA	Talaromyces sp.	99.500	0.0002441
OTU_1591	OTU_1591	Ascomycota	Trichocladium opacum	Trichocladium opacum	99.500	0.0002166
OTU_6937	OTU_6937	Glomeromycota	NA	Glomeraceae	0.000	0.0002145
OTU_1653	OTU_1653	Ascomycota	NA	Incertae sedis	100.000	0.0001704
OTU_2333	OTU_2333	Ascomycota	NA	Saccharomycetales	0.000	0.0001526
OTU_1964	OTU_1964	Ascomycota	Comoclathris	Comoclathris	99.500	0.0001152
OTU_7842	OTU_7842	Basidiomycota	Malassezia globosa	Malassezia globosa	95.025	0.0001140
OTU_1162	OTU_1162	Ascomycota	Toxicocladosporium irritans	Toxicocladosporium irritans	99.005	0.0001045
OTU_4812	OTU_4812	Ascomycota	NA	Eurotiales	100.000	0.0001037
OTU_1380	OTU_1380	Ascomycota	Plenodomus biglobosus	Plenodomus biglobosus	100.000	0.0000898
OTU_4566	OTU_4566	Ascomycota	NA	Saccharomycetales	0.000	0.0000765
OTU_234	OTU_234	Ascomycota	NA	Stachybotryaceae	98.010	0.0000581
OTU_2171	OTU_2171	Ascomycota	NA	Helotiaceae	99.000	0.0000551
OTU_351	OTU_351	Ascomycota	NA	Dothideomycetes	95.918	0.0000428
OTU_2350	OTU_2350	Basidiomycota	Malassezia restricta	Malassezia restricta	100.000	0.0000427
OTU_3690	OTU_3690	Chytridiomycota	NA	Chytridiomycota	84.878	0.0000373
OTU_314	OTU_314	Ascomycota	NA	Ascomycota	0.000	0.0000353
OTU_696	OTU_696	Ascomycota	NA	Dinemasporium sp.	97.462	0.0000277
OTU_3044	OTU_3044	Mortierellomycota	NA	Mortierella sp.	99.492	0.0000274
OTU_2983	OTU_2983	Ascomycota	NA	Helotiales	98.507	0.0000133
OTU_2805	OTU_2805	Ascomycota	NA	Pseudogymnoascus sp.	100.000	0.0000109
OTU_2881	OTU_2881	Ascomycota	NA	Arthrinium sp.	99.500	0.0000106
OTU_6435	OTU_6435	Basidiomycota	Malassezia globosa	Malassezia globosa	100.000	0.0000094
OTU_2384	OTU_2384	Basidiomycota	NA	Delicatula sp.	97.512	0.0000093
OTU_5725	OTU_5725	Mortierellomycota	Mortierella gamsii	Mortierella gamsii	99.492	0.0000091
OTU_7956	OTU_7956	Chytridiomycota	NA	Chytridiomycota	97.487	0.0000090
OTU_792	OTU_792	Ascomycota	NA	Ascomycota	0.000	0.0000075
OTU_3757	OTU_3757	Mortierellomycota	Mortierella exigua	Mortierella exigua	98.010	0.0000057
OTU_4057	OTU_4057	Ascomycota	NA	Hypocreales	96.500	0.0000050
OTU_5812	OTU_5812	Mortierellomycota	Mortierella	Mortierella	100.000	0.0000049
OTU_7623	OTU_7623	Basidiomycota	NA	Agaricales	97.500	0.0000047
OTU_1512	OTU_1512	Mortierellomycota	NA	Mortierella sp.	100.000	0.0000042
OTU_5299	OTU_5299	Ascomycota	NA	Helotiales	97.970	0.0000030
OTU_1694	OTU_1694	Ascomycota	Trichoderma atroviride	Trichoderma atroviride	100.000	0.0000029
OTU_1667	OTU_1667	Ascomycota	NA	Paraophiobolus sp.	94.500	0.0000023
OTU_3601	OTU_3601	Basidiomycota	Clitopilus prunulus	Clitopilus prunulus	100.000	0.0000021
OTU_6360	OTU_6360	Basidiomycota	NA	Agaricomycetes	96.000	0.0000019
OTU_6694	OTU_6694	Basidiomycota	Vishniacozyma dimennae	Vishniacozyma dimennae	0.000	0.0000019
OTU_2574	OTU_2574	Ascomycota	NA	Pleosporales	88.384	0.0000017
OTU_1947	OTU_1947	Ascomycota	NA	Pleosporales	100.000	0.0000016
OTU_6251	OTU_6251	Ascomycota	NA	Hypocreales	91.489	0.0000008
OTU_3169	OTU_3169	Mortierellomycota	Mortierella alpina	Mortierella alpina	100.000	0.0000006
OTU_3823	OTU_3823	NA	NA	Fungi	0.000	0.0000006
OTU_2395	OTU_2395	NA	NA	Fungi	0.000	0.0000004
OTU_3649	OTU_3649	Ascomycota	NA	Ascomycota	0.000	0.0000003
OTU_5151	OTU_5151	Chytridiomycota	Spizellomyces plurigibbosus	Spizellomyces plurigibbosus	100.000	0.0000003
OTU_7142	OTU_7142	Ascomycota	NA	Sordariales	97.990	0.0000003
OTU_3816	OTU_3816	Ascomycota	NA	Pleosporales	96.020	0.0000002
OTU_7754	OTU_7754	Ascomycota	NA	Pleosporales	95.500	0.0000002
OTU_4399	OTU_4399	Ascomycota	NA	Humicola sp.	100.000	0.0000002
OTU_5332	OTU_5332	Ascomycota	Ascobolus	Ascobolus	98.500	0.0000002
OTU_4208	OTU_4208	Ascomycota	NA	Lasiosphaeriaceae	99.497	0.0000002
OTU_4504	OTU_4504	Basidiomycota	Lepista sordida	Lepista sordida	99.000	0.0000002
OTU_7612	OTU_7612	Ascomycota	NA	Stagonospora sp.	89.394	0.0000001
OTU_7216	OTU_7216	Basidiomycota	NA	Malasseziales	99.500	0.0000001
OTU_7882	OTU_7882	Chytridiomycota	NA	Spizellomyces sp.	0.000	0.0000001
